# Host-specific leaf-mining behaviour of holometabolous insect larvae in the early Permian

**DOI:** 10.1038/s41598-025-15413-x

**Published:** 2025-08-25

**Authors:** Michael Laaß, Ludwig Luthardt, Steffen Trümper, Angelika Leipner, Norbert Hauschke, Ronny Rößler

**Affiliations:** 1 Museum für Naturkunde Chemnitz, Chemnitz, Germany; 2 TU Bergakademie Freiberg, Geological Institute, Freiberg, Germany; 3 Museum für Naturkunde Berlin, Leibniz Institute for Research on Evolution and Biodiversity, Berlin, Germany; 4 Institute of Geology and Palaeontology, University of Münster, Münster, Germany; 5 Museum am Schölerberg, Osnabrück, Germany; 6 Institute of Geosciences and Geography, Martin-Luther-Universität Halle-Wittenberg, Halle (Saale), Germany

**Keywords:** Insect-plant interaction, Leaf-mining, Endophagy, Insect metamorphosis, Holometabolan evolution, Peltaspermales, Late palaeozoic, Evolutionary developmental biology, Palaeontology, Ecology

## Abstract

Leaf-mining is a complex insect feeding behaviour that provides various advantages for reproduction success. The evolutionary origin within the holometabolan clade was hypothesised to have first occurred in the early Mesozoic. We re-examined the controversial feeding trace of *Asteronomus maeandriformis*, which is abundant in an early Permian plant fossil assemblage from central Germany. We applied multiple optical and chemical analysis techniques to unravel the distinctive morphological patterns of these insect traces. The feeding traces represent tunnels within the leaf laminae, pointing to an endophytic feeding style. In addition, we found numerous oviposition sites that exhibit a direct association with the tunnels and were most likely produced by the same organisms. At the investigated locality, more than 80% of the abundant foliage of *Autunia conferta* (Peltaspermales) was affected, indicating a host-specific mass infestation by the producer. Our results unequivocally show that endophytic feeding behaviour evolved in the holometabolan clade at least by the earliest Permian and, therefore, more than 40 Ma earlier than hypothesised. The findings reveal complex organism interactions in late Palaeozoic ecosystems that may have evolved in response to global environmental change, leading to drier conditions in the palaeotropics.

**Supplementary Information:**

The online version contains supplementary material available at 10.1038/s41598-025-15413-x.

## Introduction

Leaf-mining is a special behaviour of holometabolous insect larvae to feed tunnels inside the parenchymatous mesophyll or upper epidermis of plants. In contrast, the outer wall remains unaffected, thus shutting off mine activities from outside^1–3^. Mining occurs in leaves, cambium, flowers, fruits, and stems^2,4^.

Exophytic or endophytic oviposited eggs give rise to larvae, which form frass trails as linear, digitate, blotch or tentiform mines, among others^4^. Due to the larval ontogenetic growth, the width of mines usually increases the longer the distance from the oviposition site. Typically, leaf mines are often parallel to the leaf venation and bordered by callus tissue^1,5^. Leaf-mining is exclusively known from four holometabolous insect orders and has evolved independently in Coleoptera, Lepidoptera, Diptera, and Hymenoptera^1,3,4,6^. Phylogenetic and fossil data suggest that the early radiation of the Holometabola occurred in the Carboniferous^7–10^.

Due to the rare fossil record and the frequent absence of diagnostic features, the origin of leaf mining seems uncertain. Currently, all late Palaeozoic records of putative leaf mines have been under discussion (Table S1), including several examples from the early Permian of Thuringia, Germany^2^. Among them, there were frass trails in *Autunia conferta* peltasperm leaves^11,12^ and other pteridosperm foliage, later named *Asteronomus maeandriformis* and *A. divergens*^2^. Applying the maceration technique, it was already demonstrated in 1921 that the traces were present within plant tissue^12^ and were therefore genuine mines. Nevertheless, this interpretation was repeatedly questioned^5,13,14^. Therefore, the widespread viewpoints on leaf mining did not evolve before the Middle to Late Triassic^15–19^ or not before the Permian–Triassic boundary^20^.

Recently, the Pennsylvanian origin of leaf mining was discussed^14,21^, based on putative traces preserved on a single leaf fragment^14^. However, these structures do not provide convincing evidence as they lack typical features of leaf mines such as an oviposition site, a gradual increase in tunnel width, a terminal chamber, and coprolites.

Nevertheless, from an ecological perspective, the origin of leaf-mining behaviour might be assumed to be in the late Palaeozoic, which marks an important interval for the evolution of various organism groups and their interrelations in terrestrial ecosystems. Especially in the palaeotropical belt, a major palaeoclimatic shift is documented from various strata worldwide and linked to global pCO_2_ atmospheric changes and glaciations of the Late Palaeozoic Ice Age^22–24^. The shift led to fluctuating palaeoclimatic trends and a superimposed change from ever-wet conditions in the lower- to middle Carboniferous towards progressively drier and pronounced seasonal conditions in the upper Carboniferous and Permian^25–27^. The significant impact of this palaeoclimatic trend is documented in changes to the structure and composition of terrestrial ecosystems, which coincided with evolutionary innovations in major organism groups, including plants, vertebrates, and arthropods^28–32^. Organisms have evolved numerous new survival strategies to cope with challenging seasonal conditions, resulting in an increasing complexity of intricate ecological networks^33,34^. With regard to insect herbivory, the first occurrence of new plant clades since the upper Pennsylvanian, especially within the gymnosperms, provided new opportunities for nutrient supply^35–37^, which might have triggered co-evolutionary innovations.

Here, we reinvestigate the controversial insect feeding trace *Asteronomus*, which abundantly occurs in an early Permian fossil locality of central Germany that reflects a marginal lacustrine ecosystem that was shaped under seasonally wet conditions. The near-shore habitats were predominantly vegetated by *Autunia conferta*, a representative of the Peltaspermales, which depict a “modern” gymnosperm clade of the late Palaeozoic^36,38,39^. Using various analysis techniques, we provide evidence for *Asteronomus* representing the oldest unequivocal trace of leaf-mining behaviour. Moreover, we share insights into the producer’s biology, including host selection, larval feeding behaviour, and ontogenetic development. The main objective of our study is to clarify the nature of *Asteronomus* and to test the hypothesis whether holometabolan insect larvae were already adapted to an endophytic mode of life in the late Palaeozoic.

**Fig. 1:**
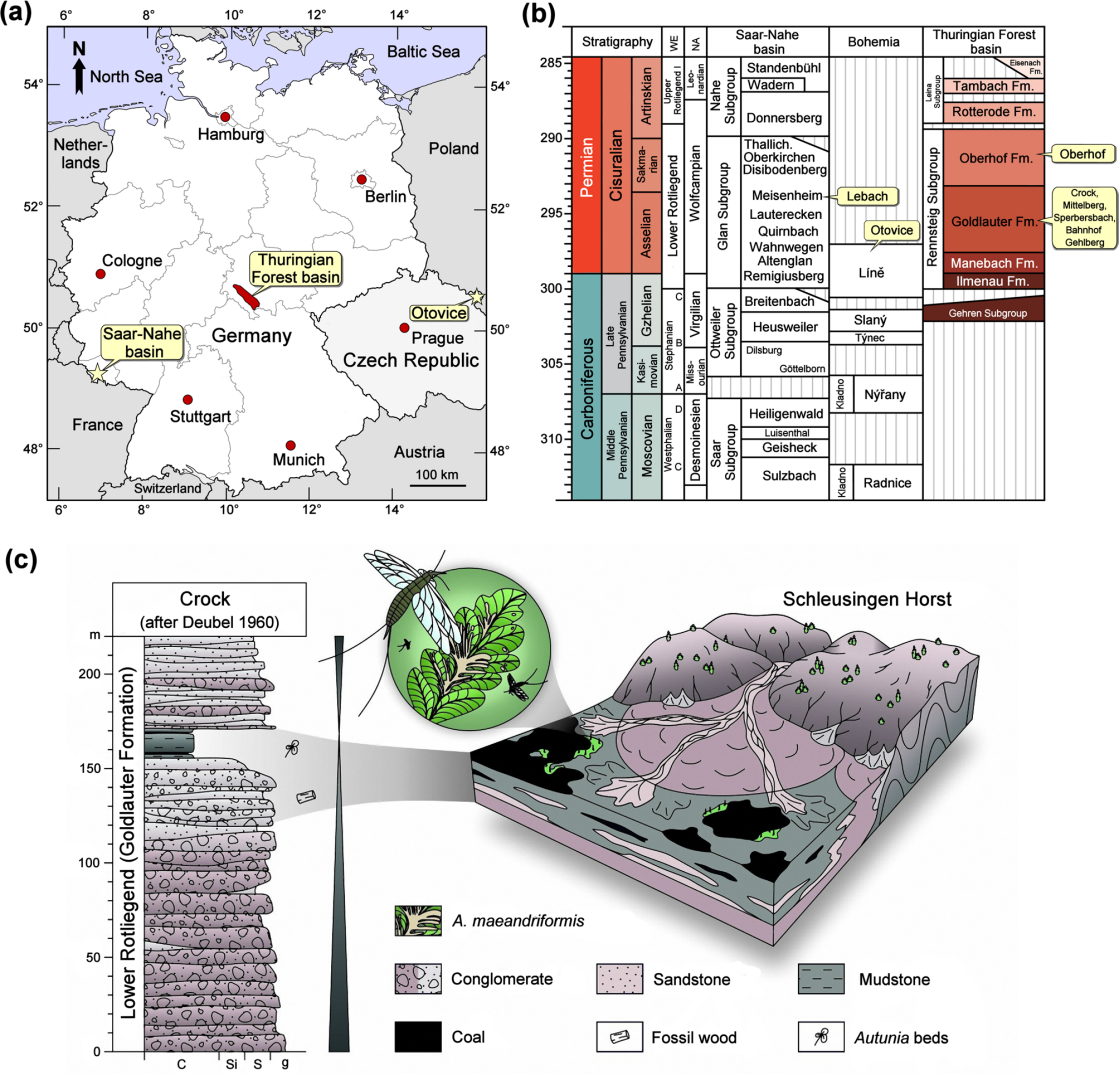
Location and stratigraphy of the locality Crock in the Thuringian Forest Basin in central Germany. **(a)** Location of Crock in Thuringia, central Germany. **(b)** Stratigraphic position of the Crock locality in the Goldlauter Formation in Thuringia, Germany. **(c)** Lithostratigraphy and depositional environment of the Crock locality. Reconstructed after^40,41^. The map in **(a)** was generated with Paint Shop Pro 7.0 (http://www.jasc.com).

## Materials and methods

### Material

All material of *Asteronomus maeandriformis* derives from the early Permian locality Crock locality in Thuringia, Germany^2^. All specimens, including thin sections and other micro-preparations are housed in the collections of the Museum für Naturkunde Berlin, the TU Bergakademie Freiberg, and the Naturhistorisches Museum Schloss Bertholdsburg in Schleusingen. For details, see Table S2.

### Age and geology of the Crock locality

Based on tectonic, lithological and palaeontological indications, Crock was correlated with the lower Goldlauter Formation of the Thuringian Forest Basin^42^ (Fig. 1a, b). This correlation is supported by palynological data, which enable interregional correlation with the Donets Basin and other early Permian occurrences^43^. Recently, a new zircon U–Pb CA–ID–TIMS age was obtained from a significant tuff of the Goldlauter Formation in the Thuringian Forest Basin: 298.4 ± 0.6 Ma^44^. The underlying Manebach Formation, also discussed to contain the Crock locality^41^, was radioisotopically constrained as 297.8 ± 2.0 to 298.4 ± 5.6 Ma based on U-Pb LA-ICP-MS ages from magmatic zircons isolated from two tuffs^45^ and as 299.1 ± 0.4 Ma based on U-Pb CA-ID-TIMS data^44^. Moreover, an early Permian age for the Crock locality is also consistent with interregional biostratigraphic correlations^46^.

**Fig. 2:**
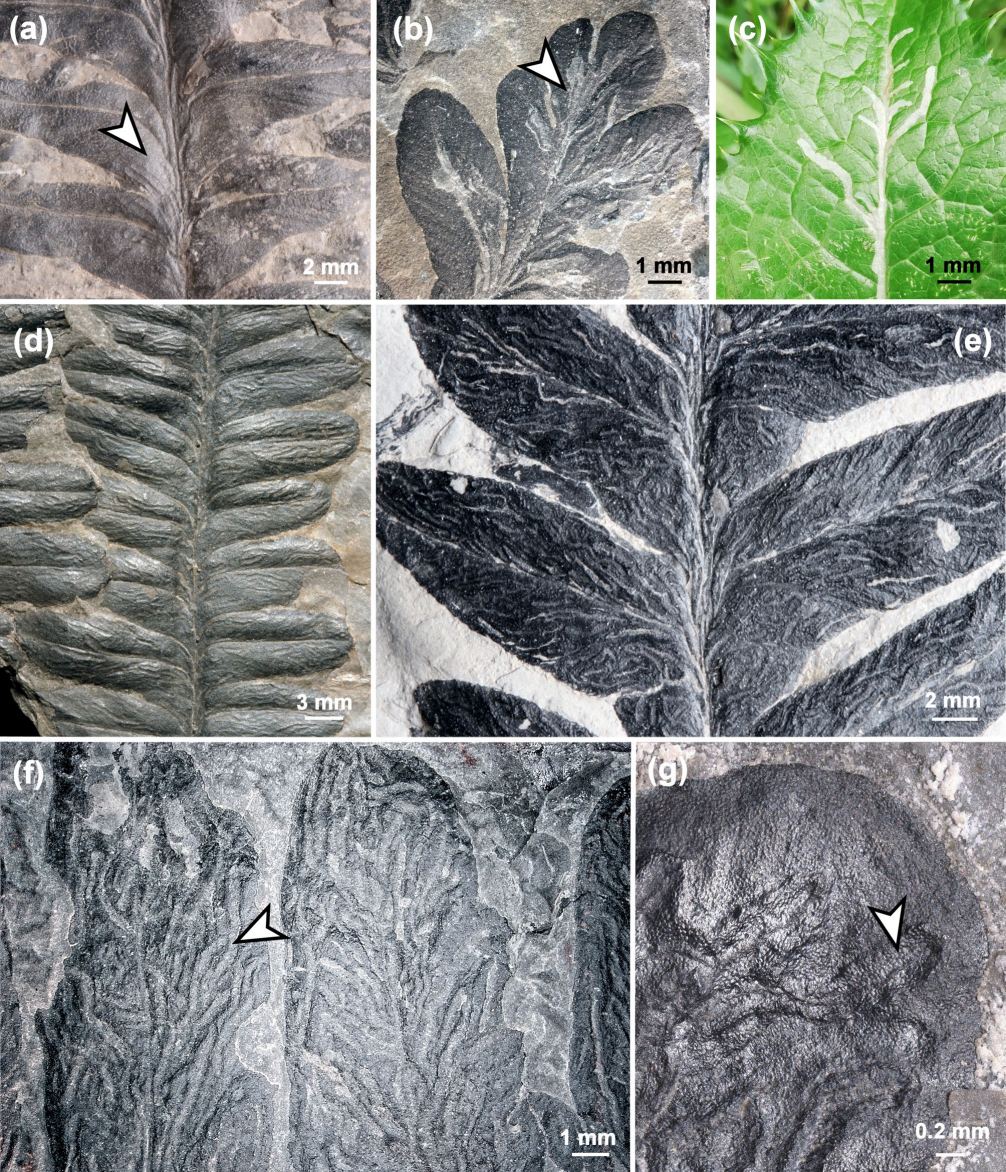
Morphology of *Asteronomus maeandriformis* in *Autunia conferta* from Crock, Thuringian Forest Basin, Germany. **(a)** *A. maeandriformis* in a star mine-like pattern on *Autunia conferta* (MB.Pb.1979/0013 **(b)** MB.Pb.1979/0015. **(c)** Recent star mines of the mining fly *Liriomyza* sp. (Diptera, Agromyzidae) on *Sonchus asper*. **(d)** Intensive damage of *Autunia conferta* by *A. maeandriformis* (FG 288/1). Note the irregular pattern compared to (A, B). **(e)** MB.Pb.1980/0517. **(f)** Holotype of *A. maeandriformis* Müller (1982) (FG 288/7) showing numerous irregular tunnels of *A. maeandriformis*. **(g)** Well-preserved upper epidermis with papillae of *Autunia conferta* covering the frass trails (NHMS AP37/5).

**Fig. 3:**
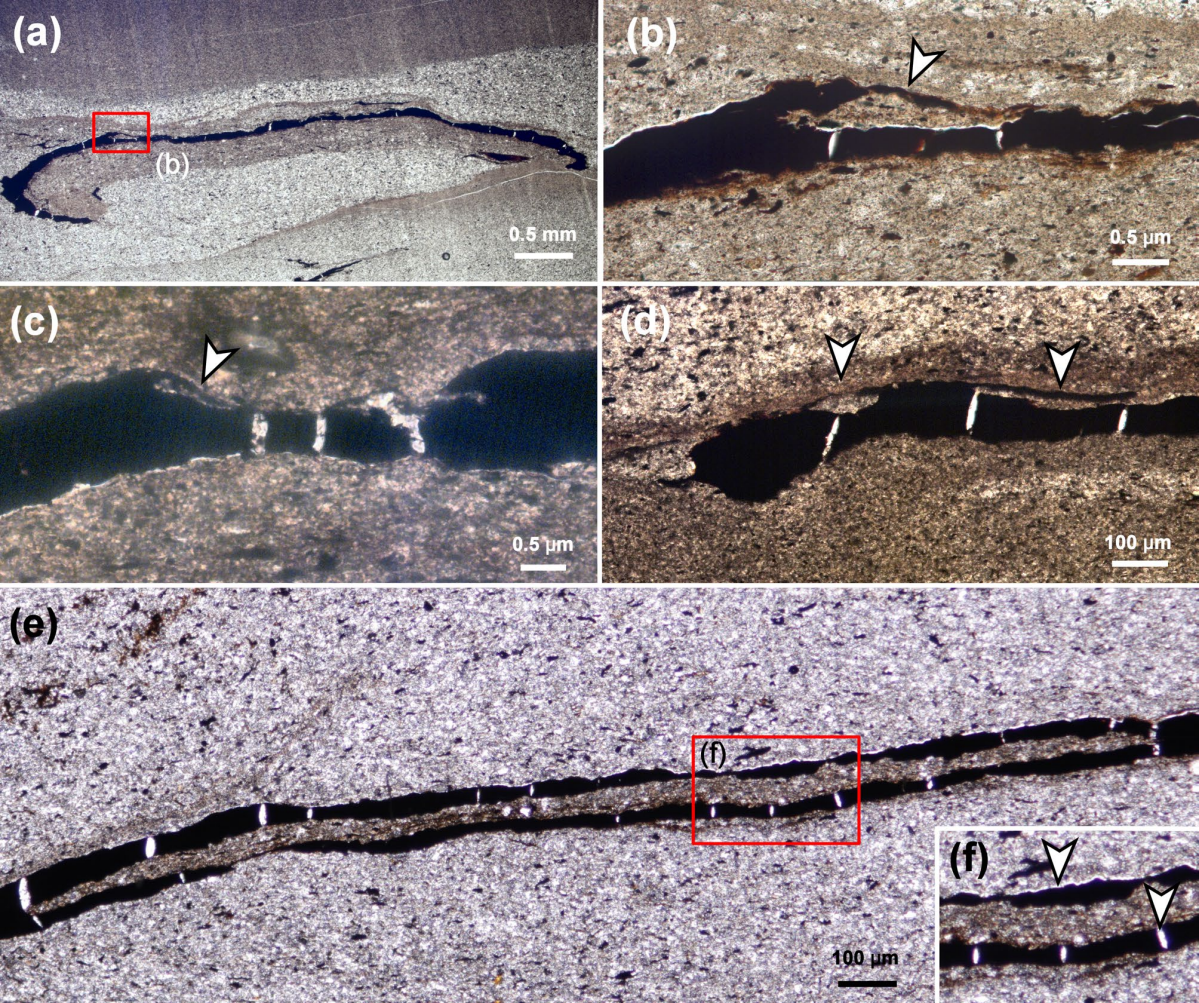
Thin sections of *Asteronomus maeandriformis* in *Autunia conferta* from Crock, Thuringian Forest Basin, Germany. **(a)** Section through an *Autunia conferta* leaf (MB.Pb.1979/0188) with frass trail (box). The scrolled margins indicate the upper side of the pinnule. **(b)** Section through the frass trail in **(a)**. Note the upper epidermis (arrow), which covers the tunnel. **(c)** Another example of a compressed tunnel in the upper parenchyma covered by the upper epidermis (arrow) (MB.Pb.1979/0179). **(d)** Transverse and longitudinal sections through endophytic frass trails in *Autunia conferta* (MB.Pb.1979/0188). **(e**,** f)** Longitudinal section through a frass trail (MB.Pb.1979/0069). Note the calcite-filled cracks and the thin calcite cover on the upper side of the leaves from shrinkage of the organic matter in (arrows in f).

The 220 m thick Crock strata formed in closely interlocked alluvial fans and peat-forming wetlands of a spatially restricted basinal branch, which flanked the Schleusingen Horst in the south-eastern Thuringian Forest Basin (Fig. S1). The basin-central type succession initially fines upsection, starting with 150 m thick, reddish to greyish alluvial psephites termed Crock Conglomerate, overlain by 6 m of greyish alluvial sandstones and 24 m thick palustrine/lacustrine deposits with two thin coal seams^40,41^ (Fig. 1c). Another 50 m thick succession of alluvial coarse-clastics follows the coal-bearing strata, concluding the succession (Fig. 1c). Plant fossils occur in an interval reaching from the upper reddish–greyish Crock Conglomerate to the fine-grained, dark-grey mudstones associated with the coal. Temporary excavations by two of the authors (RR and ST) in 2021 revealed that woody debris occurs in the upper, partly channelised deposits of the Crock Conglomerate at low concentrations. These excellently permineralized remains derived from Walchian conifers of meso- to xerophilous floras, which once inhabited the nearby basin-external uplands^47,48^. By contrast, subautochthonous leaf compressions of the peltasperm *Autunia conferta* (Sternberg) Kerp dominate the coal-related mudstones, along with a few hygrophilous floral elements such as calamitaleans and ferns^40,49^. The excellent preservation of the plant fossils in the coal-related mudstones (Fig. 2) can be explained on the one hand by the low degree of coalification^50^. On the other hand, the presence of carbonate during diagenesis^51^ likely prevented the decomposition of organic matter by neutralising humic acids, which is supported by the results of our µXRF investigation (Note S1, see also Fig. 3).

### Methods

Overview photographs of the specimens were taken by a Canon EOS 700D, a Canon EOS 450D, and a Sony Alpha 7R digital camera. The specimens were studied in detail using digital stereomicroscopes Leica S9i and Keyence VHX-6000, which were also used for microphotographs.

Thin sections were made in the preparation lab of the Museum für Naturkunde Berlin. The sections were prepared from sedimentary rocks of the Crock locality, which exhibited considerable amounts of *Autunia confert*a foliage showing distinct insect damage traces. The foliage is preserved as compressions visible as coaly relicts of the original pinnule cross-sections embedded in the fine-grained sedimentary matrix. Thin sections were ground to a thickness of approximately 100 μm and then further reduced to around 40 μm in a second step after initial documentation.

Additionally, we applied Scanning Electron Microscopy (SEM) for specimen MB.Pb.2022/2423 to identify microstructures of the chorion and larval relicts in the mines. Prior to scanning, the specimen was covered with a thin layer of gold.

Furthermore, we analysed two specimens (MB.Pb.1979/0181 and MB.Pb.1979/0182) by micro-X-ray fluorescence (µXRF) spectroscopy at the Museum für Naturkunde Berlin. For the investigations, we used a Bruker M4 TORNADO PLUS µXRF spectrometer. Further details of µXRF analysis are given in Supplementary Note 2.

Basic statistical analyses were applied to determine the variation in scar lengths and widths of representative and well-preserved oviposition scars and the widths of leaf mines. First, outliers of measured oviposition scars were excluded from the dataset following the 1.5 interquartile range (IQR) rule (for details see Fig. S8 and Supplementary Note 3). Furthermore, the dataset of oviposition scars cleaned of outliers was statistically analysed to determine variation ranges, mean values and standard deviations of the oviposition sites (Supplementary Note 3). Additionally, we measured widths of 33 leaf mines along the frass trails from distal to proximal (near the midvein) (Fig. S9).

Moreover, we investigated *A. conferta* foliage from seven early Permian localities to determine the relative abundance of leaf-mining (Table S3). The investigated material comes from the collections of the Museum für Naturkunde Berlin and Chemnitz. In all localities, *A. conferta* was a common element of the palaeoflora. Specimens were randomly selected for statistical analysis, except for those that were poorly preserved. Furthermore, we did not investigate specimens, which only showed the underside of the pinnules, because leaf mines are often only visible as shallow grooves on the upper side of the pinnules. Generally, leaf mines on *A. conferta* from Crock were much easier to identify due to their excellent preservation compared to other localities. Consequently, it cannot be completely ruled out that the content of damaged pinnae in other localities was originally slightly higher than determined here. Although such uncertain damages were not included, they are still listed in Table S3. Due to the varying degrees of fragmentation of *A. conferta*, we defined the number of undamaged and damaged pinnules per specimen to reduce the influence of taphonomy on the statistical results.

## Results

### Morphology of *Asteronomus maeandriformis*

*Asteronomus maeandriformis* usually appears as shallow grooves on the upper leaf surface of *Autunia conferta* pinnules (Fig. 2). The grooves are either arranged in a pattern resembling modern star mines (Fig. 2a–c) or irregularly with different lengths (Fig. 2d–g).

A previous study by applying cuticular analysis revealed that the grooves represent compressed tunnels in the upper parenchyma^12^. We confirm this interpretation, because our investigation provided the undamaged upper epidermis with papillae that still covered the whole leaf surface, including the tunnels (Fig. 2f). Additionally, we prepared thin sections of *Autunia conferta* foliage, which indicates the endophytic nature of the tunnels in question, in most cases situated immediately below the upper epidermis (arrows, Fig. 3a–e).

**Fig. 4:**
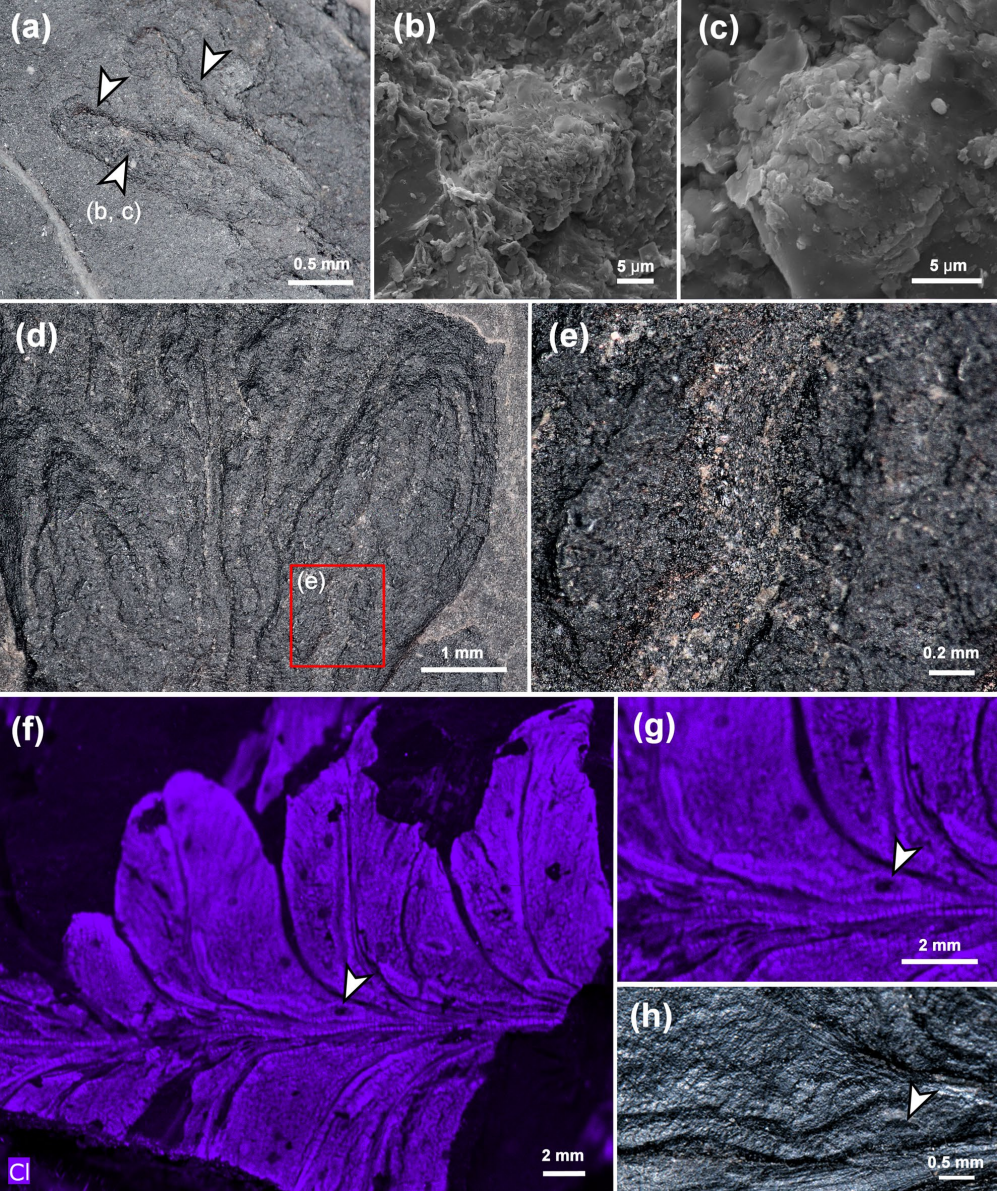
Details of *Asteronomus maeandriformis* from Crock, Thuringian Forest Basin, Germany. **(a)** Two tunnels bordered by a thickened rim of callus tissue (arrows) (MB.Pb.2022/2423). **(b**,** c)** Coprolites found at the position in the tunnel marked by the arrow in (A). **(d)** Numerous broken mines of *A. maeandriformis* showing the filling of the tunnels (holotype, FG 288/7). **(e)** Detail of a leaf mine marked by the box in **(d)**. Note the grey matrix with black components, which we interpret as frass. **(f)** Elemental map from micro-X-ray fluorescence spectrometry showing the distribution of chlorine in an *Autunia conferta* leaf (MB.Pb.1979/0181). Note the higher chlorine content in the callus tissue, which increases the visibility of the leaf mines. **(g)** Close-up of the µXRF image in **(f) **showing the putative exit hole (arrow) in **(g)**. **(h)** Photograph of (MB.Pb.1979/0181) showing the terminal part of a leaf mine with putative exit hole of the producer (arrow).

Furthermore, in the previous description, coprolites were recognised in the frass trails^2^. We investigated several tunnels using Scanning Electron Microscopy and identified coprolites with diameters of approximately 20 μm, spherical in shape, and composed of small, likely undigested components (Fig. 4a–c). However, in most tunnels, only a few coprolites were found. Other frass trails were filled with small dark components (Fig. 4d, e), which we interpret as frass. Additionally, we investigated leaf mines applying µXRF, looking for a higher content of phosphorus, as an indication of the presence of coprolites. However, phosphorus content was not significantly higher than in other leaf areas (Supplementary Notes 1, 2).

In most cases, we recognised reaction tissue at the tunnel margins (Figs. 2a, b and d–g and 6a), which shows that the damage originated during plant life. This is also supported by µXRF spectrometry (Fig. 4f, g), which detected organic-affiliated elements such as Cl and S in callus tissue (Supplementary Note S2, Figs. S2–S6). Furthermore, one of the leaf mines in an *A. conferta* pinnule revealed an exit hole near the midvein, most likely generated by the producer (Figs. 4f–h, S7).

### Oviposition sites with insect-egg remains

**Fig. 5:**
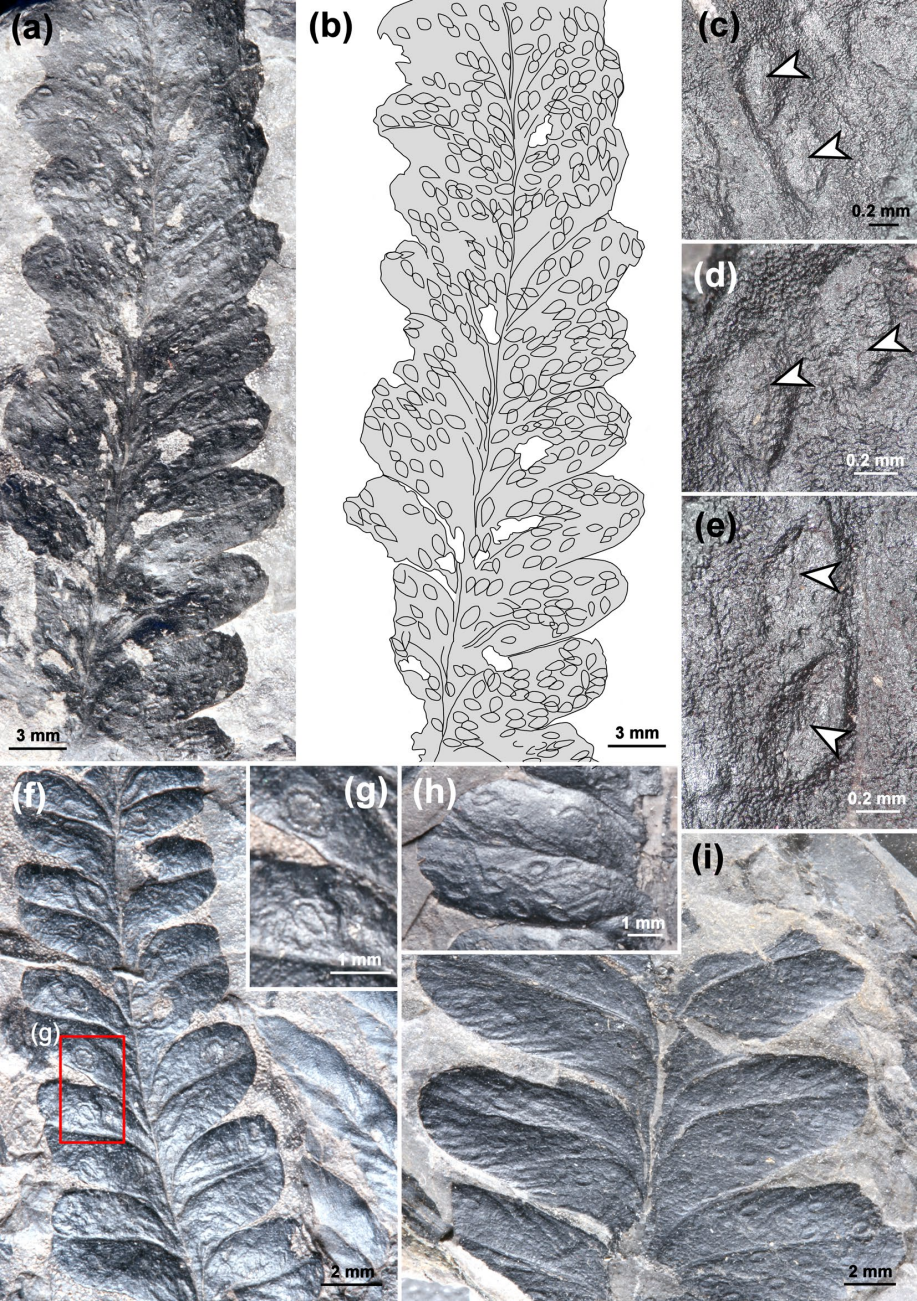
Co-occurrence of endophytic oviposition and *Asteronomus maeandriformis* in *Autunia conferta*. **(a)** Mass occurrence of oviposition scars, which likely housed unhatched eggs (NHMS Ap 37/5). **(b)** Sketch of **(a)** showing the distribution of the oviposition scars on the pinnule. **(c–e)** Oviposition scars with ovipositor slits (arrows) (NHMS Ap 37/5). **(f)** Oviposition and leaf mining on small *Autunia conferta* pinnules (MB.Pb.1979/0012). **(g)** Enlargement of the area in **(f)**. Note the callus tissue surrounding the oviposition sites. **(h)** Leaf mines and oviposition scars on *Autunia conferta* (MB.Pb.1979/0014). **(i)** Irregularly distributed oviposition scars on *Autunia conferta* (MB.Pb.1979/0020).

**Fig. 6:**
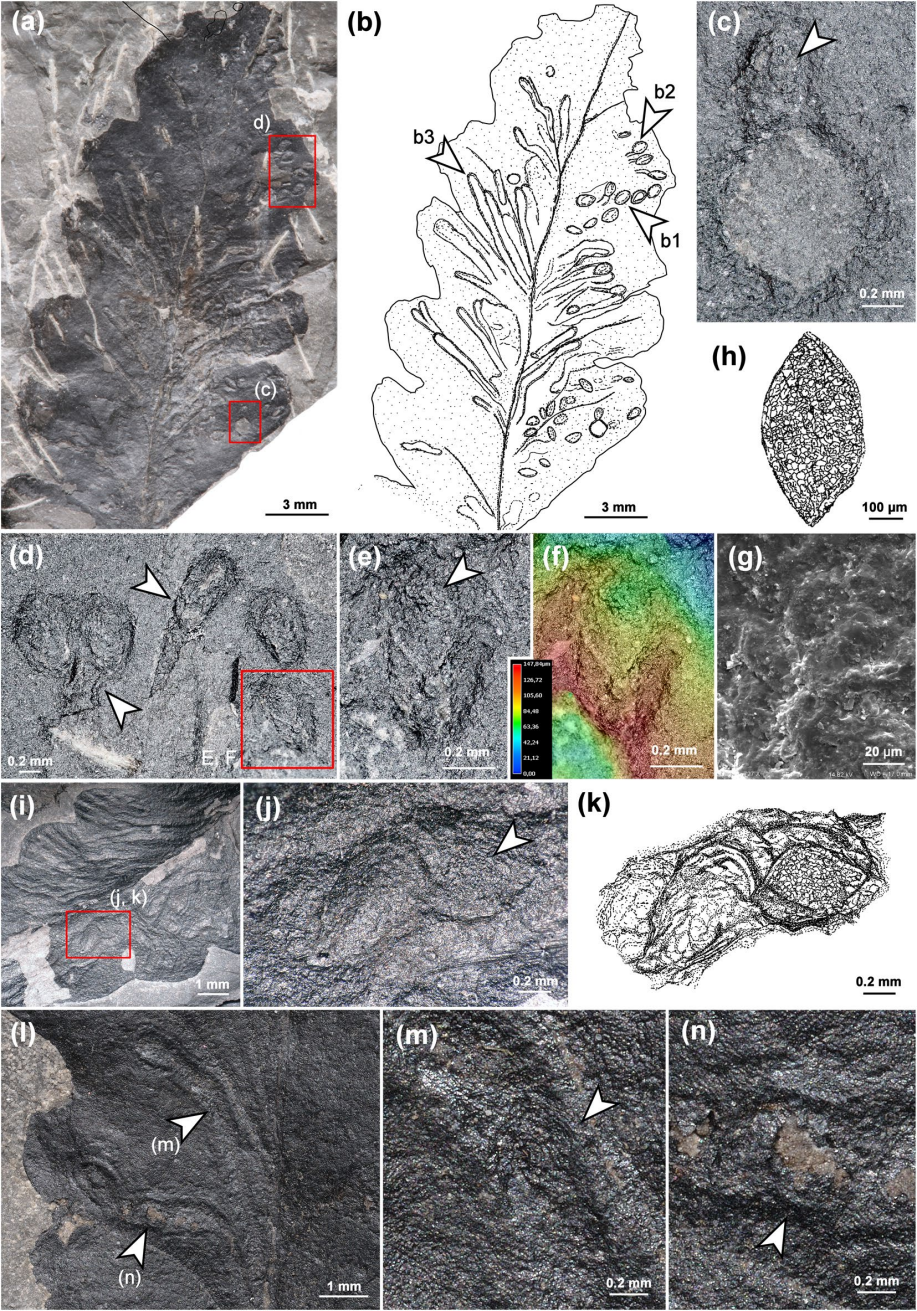
Oviposition sites with egg remains associated with *Asteronomus maeandriformis* in *Autunia conferta* from Crock, Thuringia, early Permian (Asselian). **(a**,** b)** Damaged pinnule (MB.Pb.2022/2423) showing different stages of larval activity: unhatched eggs **(b1),** oviposition with early larval activity **(b2)** and several complete leaf mines **(b3)**. **(c)** Oviposition scar with remains of an insect egg embedded in callus tissue (arrow) right next to the hole feeding. **(d)** Group of oviposition scars showing initial larval activity (arrows). **(e**,** f)** Remains of the chorion layer of an insect egg embedded in an oviposition scar. **(g)** REM micrograph of the polygonal meshwork of the chorion. **(h)** Sketch of the fossil insect egg in **(e**,** f)**. **(i)** Oviposition scars and leaf mines on *A. conferta* (FG 288/6). **(j)** Close-up of the area in **(i)** marked by a box. The right oviposition scar contains an insect egg. **(k)** Sketch of **(j)**. **(l)** Two oviposition sites with associated frass trails. (NHMS Ap-37/5). **(m**,** n)** Close-up of **(l)** showing impressions of insect eggs (arrows) inside the leaf mine (NHMS Ap-37/5).

**Fig. 7:**
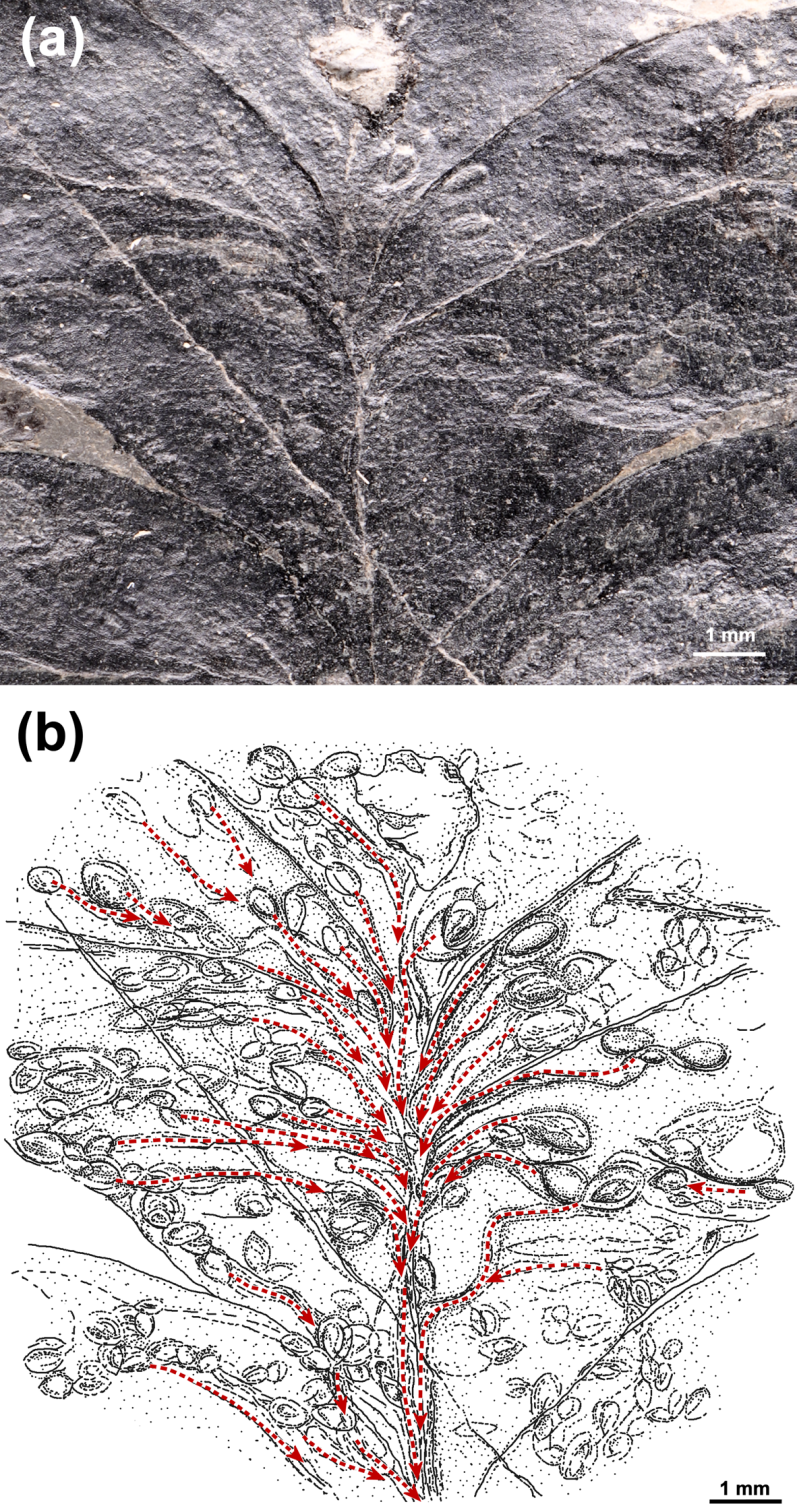
Frass pattern of *Asteronomus maeandriformis*. **(a)** Oviposition sites with associated leaf mines (MB.Pb.1979/0349). **(b)** Sketch of the frass pattern in **(a)**. Note that the frass trails originate from the oviposition sites to converge near the midvein of the leaf (red arrows).

Hitherto, oviposition sites of *Asteronomus* were unknown and supposed to be near the midvein of the leaves^2^. We recognised numerous *A. conferta* specimens with endophytic oviposition scars (Figs. 5, 6 and 7). The oviposition scars are small, lenticular, often parallel to the venation, and irregularly distributed on the pinnules. In places, even ovipositor slits are preserved (Fig. 5c–e). In many cases, the oviposition sites are associated with the leaf mines (Figs. 6d and l and 7). Scar dimensions range between 0.6 and 1.2 mm (mean 0.9 mm) in length and 0.3 to 0.8 mm (mean 0.5 mm) in width (Fig. S8a, Supplementary Note S3).

We even identified insect-egg remains in some broken oviposition scars (Fig. 6d–n). The eggs are 0.5 to 0.8 mm long and 0.3 mm to 0.6 mm wide (Fig. S8a). The eggs’ aspect ratio, defined by the quotient between length and width^52^, is approximately 1.7. The chorion reveals a polygonal meshwork (Fig. 6e–k).

### Frass pattern of *Asteronomus*

The well-preserved specimen illustrated in Fig. 7 exhibits the *Asteronomus* frass pattern, which resembles modern star mines (asteronomes) (Fig. 2c). Accordingly, the producers initiated their frass trails from randomly distributed oviposition sites on the leaf surface. In most cases, the tunnels followed the venation to converge in the midvein region (Fig. 7b). This is also supported by the widths of the frass trails, which increase towards the midvein due to the ontogenetic growth of the producer (Fig. S9). Our measurements revealed tunnel widths of 0.19 to 0.65 mm (mean 0.36 mm) proximally, near the oviposition sites and 0.21 to 0.78 mm (mean 0.47 mm) distally, near the midvein. However, in some cases, leaf mines show positive and negative excursions of mine width (Figs. S7, S9). Width variations of leaf mine width in Fig. S7 may be caused by preservation bias, varying nutrient density, toxicity and different development of plant tissue and the ethology of the producers. Possible explanations for a negative trend of mine width towards the midvein in Fig. S9 could be (1) that larvae feed proximally in deeper plant tissue resulting in less visible impressions on the leaf surface, (2) differences in development of reaction tissue along the frass trail, (3) changes in feeding direction in rare cases or (4) other larvae or oribatid mites entered the tunnel and fed in it.

The irregular frass pattern mentioned earlier (Figs. 2d–f and 4d) was, in all likelihood, produced by the same culprit, as we found smooth transitions between the two patterns. The most likely explanation is that the frass pattern depends on the number of producers per pinnule. If only a few producers feed on a pinnule, a regular pattern (asteronomes) results. In the case of many individuals in a relatively small pinnule area, almost the entire parenchymatous mesophyll was exploited and consumed, resulting in irregularly distributed tunnels of different lengths (Figs. 2d–f and 4d).

## Discussion

### Interpretation of* Asteronomus maeandriformis*

Our material meets the standard criteria of leaf mines^1^, according to which leaf mines are endophytic frass trails in living plant tissue (1) and bordered by callus tissue (2). Leaf mines originate from exo- or endophytic oviposition sites (3). Typically, but not always, the leaf mines may contain coprolites (4), and the width of the leaf mines increases along the trail due to the ontogenetic growth of the larvae (5). Finally, leaf mines terminate in a terminal chamber, or the larvae leave via an exit hole (6).

As shown, there are several lines of evidence in favour of the endophytic nature of *Asteronomus* during the plant’s life. Furthermore, we identified discernible oviposition sites (Figs. 6 and 7), evidence of a gradual increase in mine width (Fig. S9), and, in some cases, escape structures of the producers (Fig. 4f–h). The low content of coprolites and the low level of phosphorus in the trails do not rule out the interpretation as leaf mines because not all leaf miners leave coprolites in the tunnels. For example, modern sap-feeding larvae only produce fluid excreta. Lepidoptera, and some Diptera remove their coprolites from the mines to reduce risk of bacterial infection from decomposition of faecal pellets. For example, caterpillars produce slits or holes for ejection of the coprolites^1^. In some cases, well-preserved mines from Crock show longitudinal slits which might be used for removal of coprolites (Fig. 2g). Sap-feeders almost exclusively consume liquid contents of the plant. However, they bite through solid walls in the plant tissue but don´t consume them and therefore do not excrete solid pellets^1^. In any case, it seems reasonable that the producers of the leaf mines from Crock also developed a strategy to avoid bacterial infection from faecal pellets, probably by removing the coprolites or by avoiding solid plant tissue as nutrition.

### Host plant

At Crock, *Asteronomus maeandriformis* occurred in the peltasperm *Autunia conferta*. The host plant of the second ichnospecies, *Asteronomus divergens*, is the medullosan pteridosperm *Barthelopteris germarii* (Giebel) Zodrow, Shute et Cleal, which is well recognisable because of its anatomising venation. This fossil-species is also well adapted to stressful environmental conditions^53^.

A possible explanation for the preference of pteridosperms as host plants might be given by the leaf anatomy, which often possessed thicker and larger pinnules than other late Palaeozoic plants^39,54–56^. The leaf mines were ca. 0.3 mm wide, but only 0.1 mm thick, which is a result of diagenetic compression (Fig. 3). Originally, a minimal cuticle thickness of 0.3 to 0.4 mm was necessary to house frass tunnels of *Asteronomus*, which was probably only possible in pteridosperm foliage. In particular, the thickness of the mesophyll is essential for leaf miners because they prefer nutrient-rich, internal mesophyll cells and usually avoid epidermis and/or vascular tissues^4^.

### Potential producers of *Asteronomus*

There are three lines of evidence to identify the most likely producer of leaf mines: (1) comparisons of the fossil leaf mines with those of modern leaf-miners, (2) consideration of phylogenetic data about the origin of potential leaf-mining insect groups, and (3) the occurrence of the suspected leaf-mining insect groups in the same deposits in which the leaf mines were documented^17^. In this study, the morphology of the insect eggs and coprolites represents additional valuable sources of information. However, comparisons between present-day and fossil behaviour are limited, as egg-laying and leaf-mining behaviour may have evolved several times convergently in different groups. Furthermore, the ichnofossils date from a time when the insect fauna differed significantly from today’s.

Previous studies^13^ proposed leaf-mining mites as possible producers of *Asteronomus maeandriformis*, because oribatid mites are known to have bored tunnels in Palaeozoic and Mesozoic plant tissues^5,13^. These arthropods can be excluded as producers of *Asteronomus* because endophytic oviposition is unknown from mites^57^.

In modern insects, endophagous feeding modes occur in Condylognatha (Thysanoptera and Hemiptera) and Holometabola^4^. However, leaf mining is unknown from Thysanoptera and Hemiptera. This is because these orders undergo an incomplete (hemimetabolous) metamorphosis, in which the immature stages retain the same feeding form as the adults. As a result, the diversification of endophagy is limited to only a few feeding modes and constrains the larvae from feeding in the parenchyma of plants^4,6^. Furthermore, Thysanoptera and Hemiptera are unable to bore into plant tissue due to their sucking mouthparts^4^. Therefore, it seems very unlikely that *a member of the Condylognatha produced Asteronomus*.

In contrast, holometabolous insects undergo complete metamorphosis, which is accompanied by drastic changes in the body structures of larvae, pupae, and adults. This morphological plasticity during ontogeny enabled holometabolous insects to evolve tiny, grub-like larvae without large appendages – an essential adaptation for living in narrow tunnels inside plant tissues^4,6^. Today, leaf mining is restricted to Hymenoptera, Diptera, Coleoptera and Lepidoptera^1,3,4,6^. Moreover, endophytic oviposition is also a common reproductive strategy among holometabolous leaf miners.

Several phylogenetic studies predict that all leaf-mining holometabolous insect orders may have originated in the Carboniferous^10,58,59^. Accordingly, stem-Hymenoptera evolved between the Early Mississippian and the Mid-Pennsylvanian; stem-Diptera and Coleoptera in the Pennsylvanian. This evolutionary development is also supported by fossil evidence^8,9,60^. Fossil Lepidoptera were first documented from the Triassic–Jurassic transition^58,61^, but phylogenetic data suggest that the oldest members of the Lepidoptera crown group already appeared in the Pennsylvanian^59^.

Although only a few fossil records exist, a remarkable diversity of feeding strategies has already been proposed for Pennsylvanian holometabolous larvae^8^. Among them, there were externally feeding eruciform larvae, characterised by a caterpillar-like body plan, downwardly directed mouthparts and abdominal leglets^8,60^. The only fossil records of this type are *Srokalarva berthei* from the Middle Pennsylvanian (Moscovian) of Mazon Creek, USA^8,60^ and *Metabolarva bella* from the Middle Pennsylvanian of the Piesberg in northern Germany^9,60^.

A second type is campodeiform holometabolous larvae. Only one specimen of uncertain affinities with legs and long caudal filaments was described as *Cavalarva caudata* from the Permian of Russia^62^.

Regarding the origins of leaf mining, the third type, eruciform holometabolous larvae, is of special interest, because these larvae possess a legless, grub-like body form – an adaptation to live in narrow tunnels inside plant tissue. Eruciform larvae are documented from the complexly constructed borings *Pectichnus multicylindricus* in late Pennsylvanian and Permian conifer wood, which contained larval and adult mandibles and other elements representing multiple developmental stages (instars) of wood-boring beetles^63,64^.

The fossil record of the palaeo-entomofauna at the key locality Crock is restricted to several Blattoid insect wings^42,65^. Only two roachoid species were reported: *Anthracoblattina* and *Phyloblatta flabellata*^42,65^. The hitherto only indication for the presence of Holometabola at Crock is wood and its callus-producing reactions, as well as the borings produced by beetle larvae, *Pectichnus multicylindricus*^64,66^.

Another line of evidence to identify the most likely producer of fossil leaf mines is comparing the frass pattern with those of modern ones, which is usually species-specific^4,17^. Interestingly, *A. maeandriformis* resembles the mining patterns of the dipteran families Agromyzidae, Anthomyiidae and Drosophilidae^1,67^, which produce frass trails along the midvein with radial offshoots (Fig. 2c). The leaf mines of Agromyzidae are narrow, often irregular serpentine frass trails that abruptly change their direction and sometimes overlap with earlier traces^67^. The larvae of *Liriomyza* prefer to feed just below the upper surface of the leaf, in the palisade parenchyma. The eggs of Agromyzidae are also inserted endophytically. Oviposition marks and eggs of Agromyzidae have similar dimensions, i.e. 0.5 mm and 0.25 mm in diameter, respectively^67^. Interestingly, a stereotypic behaviour was reported in *Liriomyza*, which feeds leaking sap after penetrating the leaf with the ovipositor^68^. In this way, they produce feeding punctures or small holes, which occur with the mines on the same leaves^67,68^. Piercing and sucking punctures, bordered by a rim of reaction tissue, also occur together with *Asteronomus* on *Autunia conferta* (Fig. S10b–d). Notably, other punctures, not bordered by reaction tissue and often preserved as small elevations, are also abundant on pteridosperm foliage (Fig. S10e). Investigation of extracted cuticles showed that the spherical bodies were secretory cavities^69^. Due to the presence of reaction tissue around the punctures from Crock, we are sure the structures resulted from damage caused by sap-feeding insects. The low content of coprolites in the mines might be an indication of sap-feeding larvae. If this is correct, it would also be an argument that the same producer caused the piercing and sucking damages. Nevertheless, the witness whether the punctures and leaf mines go back to the same producer remains challenging.

It should be noted here that caution is still necessary in assigning extant producers to fossil leaf mines, because reproductive strategies and feeding behaviour may have evolved several times convergently. Furthermore, the fossil record of early holometabolous insects, particularly holometabolous larvae, is scarce, and we cannot exclude that extinct insect lineages produced patterns like those of modern insects.

The morphology of the insect eggs may also provide additional information about the producer. Fossil insect eggs are rare due to their low potential for fossilisation. The most resistant component is the outer layer of the eggs, the chorion, which protects the embryo mechanically and mediates the flux of gases and water between the embryo and the environment^70,71^. Therefore, comparisons of the eggs with other fossil eggs are limited to only a few specimens: The earliest remains of holometabolous insect eggs come from the beetles, which produced the borings named ichnospecies *Pectichnus multicylindricus* in the late Permian conifer wood *Ningxiaites specialis*^63^. Interestingly, the egg chorion also revealed a reticulate polygonal meshwork^63^, which suggests that this feature already existed in early Holometabola. Additionally, fossil insect eggs are only known from the late Triassic^72,73^, Meso- and Cenozoic^74^. Another occurrence of a polygonal chorionic meshwork is reported from the Cretaceous insect eggs *Transpiroveon polygonatum* of uncertain affinities^75^. Due to the simple construction, the latter authors interpreted it as a primitive intrachorionic transpiration system. However, polygonal patterns of the chorion are found in many modern insect groups, such as Odonata, Ephemeroptera, Hemiptera, Diptera, and Coleoptera^71,76^. Consequently, this feature is not diagnostic for a specific producer. The same holds true for the eggs’ size, shape, and aspect ratio. Symmetrical, lenticular, uncurved eggs are also known from many insect groups^52,77^.

Finally, the size and shape of coprolites may be indicative of some groups of producers^78^. The size of the coprolites in the tunnels is very small and falls into the range of small insect larvae and oribatid mites (Fig. 4b, c). Although oribatid mites can be excluded as producers of *Asteronomus* due to their different oviposition strategies, it cannot be completely ruled out that they revisited the frass trails and might have produced small coprolites.

In summary, we conclude that the most likely producer of *Asteronomus maeandriformis* was a member of the Holometabola.

### Mass occurrence of *A. maeandriformis* at Crock locality

**Fig. 8:**
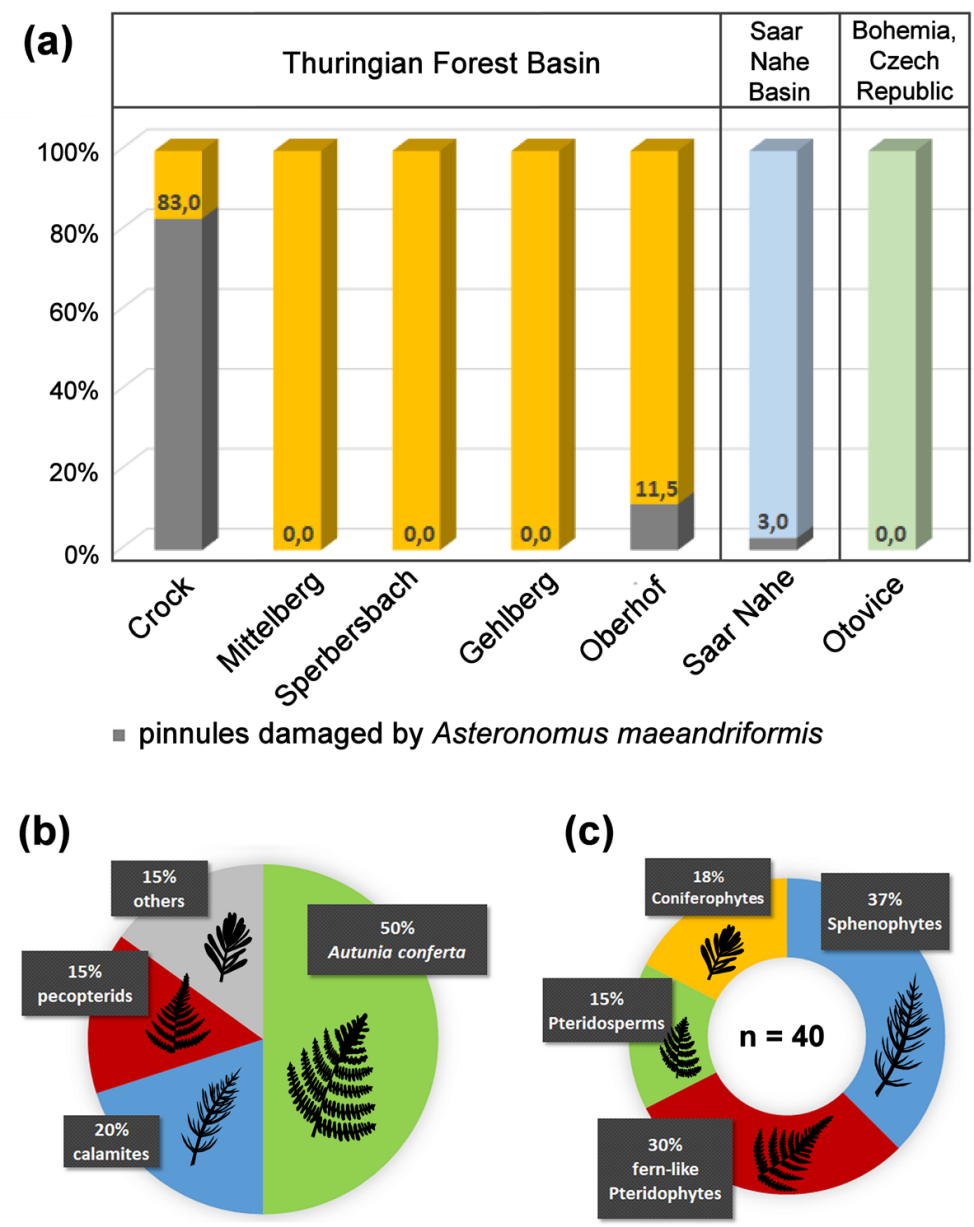
Size of oviposition scars, width of *Asteronomus maeandriformis*, and abundance of leaf-mining at Crock compared with other localities. **(a)** Relative abundance of *A. maeandriformis* on *Autunia conferta* at different early Permian localities. Note the high content of damaged *Autunia* foliage by *Asteronomus* mines at Crock, Thuringia. **(b)** The abundance of plant groups at Crock. **(c)** Plant diversity at Crock. n, number of species. Data in **(b, c)** from^42^.

Our statistical analysis revealed that 83% of *Autunia conferta* pinnules were damaged by *Asteronomus maeandriformis* at Crock, which contrasts distinctly with other early Permian sites, where leaf mines were either completely absent or rare (Fig. 8a, Table S3).

Due to the lack of outcrop data, the lithostratigraphic context of the historically collected Crock material is unclear in detail. Therefore, questions concerning the temporal aspect of ecosystem development remain. The *A. conferta*-bearing strata consist of fine-grained, and most likely bioturbated, mudstone, suggesting low sedimentation rates under long-term, moisture-loving conditions. In addition, *A. conferta* remains are found in various stacked laminae, implicating an extended duration under stable habitat conditions, at least at a scale of decades. Independent of the temporal aspect, the striking abundance of damaged *A. conferta* pinnules at Crock strongly suggests a local mass occurrence of leaf mining insects.

Generally, population dynamics in ecosystems may be influenced by several biotic and abiotic (environmental) factors^79^. Biotic factors include: (1) intraspecific competition for food and other resources, (2) social stress between individuals, (3) the number of predators, and (4) illness and parasitism. Abiotic factors are, among others, (1) environmental conditions such as temperature, light and water availability, (2) soil characteristics, (3) extreme events such as floods, fires, drought periods, volcanic activity, and (4) interspecific competition. Notably, some taxa are characterised by cyclic population dynamics^79^.

Remarkably, extreme variations in frond morphology and pinnule size among *A. conferta* leaves were recognised at Crock^39,49^, which might be an indication of fluctuating environmental conditions. It seems reasonable that rough environmental conditions had an impact on the vitality and defence mechanisms of *A. conferta* against leaf miners. Otherwise, it is also possible that the stunted growth of small *A. conferta* fronds was primarily the result of damage by insect larvae.

Currently, the most convincing explanation for the mass occurrence of *Asteronomus* at Crock is the exhaustive food source for the leaf miners because *A. conferta* was by far the most abundant plant in the palustrine/lacustrine facies of Crock, reaching an amount of 50%, followed by 20% calamitaleans and 15% tree ferns with pecopterid foliage^42^ (Fig. 8b). This striking abundance of *A. conferta*, which contrasts with other early Permian localities^42^, was undoubtedly an essential prerequisite for the mass emergence of leaf miners.

### Palaeoecology and palaeoenvironment of Crock

The sequence at Crock is interpreted as an infill of a small Rotliegend basin with conglomerates at the base, overlain by alluvial and palustrine/lacustrine deposits with small coal seams at the top (Fig. 1c). The damaged *A. conferta* material derives from the palustrine/lacustrine facies, which is supported by the co-occurrence of freshwater bivalves (Anthracosia), branchiopods (conchostraca) and ostracods^42,51^. The fine clastic sediments, the peat formation, as well as the occurrence of large separate *A. conferta* fronds together with their in-situ root systems, suggest at least a sub-autochthonous plant community, which most likely inhabited the shallow-water shoreline of a lacustrine/palustrine palaeoenvironment^39,42,51^.

Generally, seed ferns inhabited various areas, including tropical ecosystems, wetlands, and seasonally dry habitats^80,81^. This was also reported for several peltasperm species, such as *Autunia conferta* and *Autunia naumannii*; both are obviously mutually exclusive at the same locations^45^. Interestingly, the co-occurrence of *A. conferta* with peat-forming plants, such as calamitaleans and pecopterids, in the wet palustrine habitat at Crock is quite exceptional, because *Autunia* is a plant that usually occurred in non-peat-forming mesophilous vegetations^39^. Possible explanations are that *Autunia* migrated into the habitat of peat-forming plants reacting to falling groundwater levels or occupied a vacant ecological niche after the decline of peat-forming plant communities in the early Permian^39,42^.

Generally, the palaeoflora of Crock revealed a lower diversity than other early Permian localities in the Thuringian Forest Basin^42,50^ (Fig. 8c). For example, the medullosan seed ferns, some marattialean tree ferns and cordaitaleans are largely absent.

### Implications for the early evolution of the Holometabola

The late Palaeozoic floral change in the palaeotropical latitudes of Euramerican Pangea coincides with a long-term global change from wet conditions in the Middle Pennsylvanian to pronounced seasonally dry conditions since the early Permian^82–84^. The seasonally dry palaeoclimatic conditions in the intramontane ecosystems of central Europe but also of the lowlands in North America seem to have favoured the radiation and further development of seed plants, encompassing early gymnosperms, such as diverse coniferophytes, ginkgophytes and cycadophytes but also some pteridosperm clades such as the Peltaspermales, including *A. conferta*^28^. The dominance of *Autunia* at Crock provides an example of how successful these new plant clades were in occupying particular ecological niches, likely due to an efficient reproductive strategy in combination with increased plasticity, referred to as ecological adaptations^39,49^. The radiation of new plant clades provides the basis for co-evolutionary processes in mutualistic organisms, such as arthropods and insects. Mass occurrences of specific plant taxa might have resulted in specific adaptive feeding patterns and an extensive spread of the corresponding organisms.

Concurrently, insects that utilised *A. conferta* and other pteridosperms as host plants had to overcome several environmental challenges. Particularly, insect eggs and small, immature stages are most vulnerable to water loss^4,85^. Endophytic oviposition and leaf mining create a relatively stable microclimate within water-saturated plant tissue during early ontogenetic stages, providing protection against desiccation, flooding, solar radiation, and temperature fluctuations. Likewise, the inner tissue of leaves is an attractive, almost unlimited food source, and the larvae are better protected inside plants against predators and parasitoids^3,4,84,86^. A further benefit of an endophytic mode of life is the lower risk that eggs or larvae lose contact with the host plant^4^.

Endophytic oviposition is as old as leaf mining. The earliest evidence comes from the late Bashkirian of the Donets Basin, Ukraine^87^. The strategy of laying eggs with an ovipositor in plant tissue likely first evolved in ancient aquatic insect groups such as the Odonatoptera and Palaeodictyoptera^88^. However, these insects were still largely dependent on the proximity of water due to their aquatic or semiaquatic larvae^32^. In the late Palaeozoic, endophytic oviposition became a successful and widespread behaviour that occurred in diverse Palaeozoic insect groups, such as Palaeodictyoptera, Odonatoptera, Dictyoptera, Archaeorthoptera, and Hemipteroidea^88,89^.

Concerning the trend of aridification from the Middle Pennsylvanian to early Permian, endophytic oviposition in combination with leaf-mining became beneficial adaptations for holometabolous insects because they reduced their water dependence during early ontogenetic stages and enabled them to colonise seasonally drier habitats.

Finally, our results show that the endophytic mode of life already evolved in the holometabolous clade at least in the earliest Permian, and thus, more than 40 million years earlier than hypothesised. This complex behaviour might have evolved in response to global environmental change towards drier conditions in the palaeotropics.

## Supplementary Information

Below is the link to the electronic supplementary material.

Supplementary Material 1

## Data Availability

Data is provided within the manuscript or supplementary information files.
