# Supplementary material for: Host-specific leaf-mining behaviour of holometabolous insect larvae in the early Permian
Source: Sci Rep. 2025 Aug 25;15:31241. doi: 10.1038/s41598-025-15413-x (PMC12378220; doi:10.1038/s41598-025-15413-x)
Supplement: Supplementary file 1 — Supplementary Material 1 [file 41598_2025_15413_MOESM1_ESM.docx]

## Supplementary Material

**Article title:** Host-specific leaf-mining behaviour of holometabolan insect larvae in the early Permian

**Authors:** Michael Laaß, Ludwig Luthardt, Steffen Trümper, Angelika Leipner, Norbert Hauschke, Ronny Rößler

**Supplementary Table S1.** Selected late Palaeozoic to Triassic damages interpreted as possible leaf mines.

| **Stratigraphy** | **Ichno-species** | **Putative producer** | **Host plant** | **Localities** | **References** |
| --- | --- | --- | --- | --- | --- |
| Middle Pennsylvanian (Moscovian) | uncertain leaf mine | probably holometa-bolous insects | *Macroneuropteris scheuchzeri* (Medullosales) | Rhode Island Fm. of Massachusetts, USA | [1] |
| Pennsylvanian (Moscovian) and late Permian (Wuchiapingian) | *Cuniculonomus (Acrophio-nomus) simplex* | – | *Neurocallipteris subauriculata, Taeniopteris eckardtii* (pteridosperm) | Zwickau, Saxony and Mansfeld, Saxony-Anhalt Germany | [2,3] |
| Pennsylvanian (Moscovian) | *Cuniculonomus (Acrophio-nomus?) subtilis* | – | *Neurocallipteris britannica* (pteridosperm) | Zwickau, Saxony, Germany | [2] |
| Pennsylvanian (Gzhelian) | possible irregular mines | – | pteridophyte or pteridosperm rhachis | northern Iberian Peninsula (León), Spain | [4] |
| Pennsylvanian (Gzhelian) | *Asteronomus* isp. | – | *Neurodontopteris auriculata*  (Medullosales) | Wettin, Saale Basin, Germany | [5] |
| Late Pennsylvanian (Gzehlian) – early Permian (Sakmarian) | *Cuniculonomus undulatus* | – | *Odontopteris* sp. (Pterido-sperma) | Plötz near Halle, Wettin Member and Oberhof Fm., Thuringia, Germany | [2,3] |
| Permian (Sakmarian) | *Mixophytonomus atavus* | probably Coleoptera | *Cordaites* stem | Blauer Stein between Schmücke and Stützerbach, Manebach Fm., Thuringia, Germany | [2,6] |
| Permian (Asselian) | *Asteronomus divergens* | Holometa-bolan insects | *Barthelopteris germarii*  (Medullosales) | Homigtal near Breitenbach, Goldlauter Fm. Thuringia, Germany | [2,6], this paper |

| **Stratigraphy** | **Ichno-species** | **Putative producer** | **Host plant** | **Localities** | **References** |
| --- | --- | --- | --- | --- | --- |
| Permian (Asselian) | *Asteronomus maeandriformis* | Holometa-bolan insects | *Autunia conferta* (Peltasper-males) | Crock, Goldlauter Fm. Thuringia, Germany | [2,3,6–8], this paper |
| Permian (Sakmarian) | *Cuniculonomus tenuis* | Holometa-bolan insects, probably Coleoptera | *Odontopteris lingulata* (pteridosperm) | Lochbrunnen near Oberhof, Oberhof Fm., Thuringia, Germany | [2,6] |
| Late Pennsylvanian – late Permian (Wuchuapingian) | possible U-shaped leaf mines *(Cuniculo-nomus simplex)* | – | *Taeniopteris eckardti* | Zwickau and Mansfeld, Germany | [2,3,9] |
| Permian (Sakmarian or Artinskian) | possible bifurcating leaf mine (DTX) | Coleoptera | *Glossopteris* cf. *indica* | Morro do Papaléo Mine, State of Rio Grande do Sul, Brazil | [10] |
| Permian (Sakmarian or Artinskian) | possible linear mine (DT99) | – | *Glossopteris communis* | Morro do Papaléo Mine, State of Rio Grande do Sul, Brazil | [11] |
| Permian | possible blotch mines | – | *Glossopteris* sp. | La Golondrina Fm., Santa Cruz Province, Argentina | [12] |
| early Permian | coprolites in mesophyll of marginal areas of fern pinnules | probably oribatid mites | undetermined fern frond | Wuda Coalfield, Inner Mongolia, China | [13] |
| late Permian – Early Triassic | curvilinear mines | Coleoptera | *Vjaznikopteris rigida* (Peltasper-males) | Volga River Basin, Russia | [14] |
| Late Triassic (late Norian–early Rhaetian) | serpentine trace | Nepticulid (Lepidop-tera) | gymnosperm leaf | Caturrita Fm., Brazil | [15] |

| **Stratigraphy** | **Ichno-species** | **Putative producer** | **Host plant** | **Localities** | **References** |
| --- | --- | --- | --- | --- | --- |
| Middle Triassic | serpentine leave mines (DT41) | – | *Scytophyllum bergeri* (pteridosperm) | Anisian Furkelpass and related floras, Richthofen Fm. near Olang/Valdaora, northern Dolomites, and Monte Agnello Flora, Vulcanites near Predazzo, central Dolomites, Italy | [16] |
| Middle Triassic (Anisian) | thread-like leaf mine (DT71) | – | “*Pterophyllum*” (cycadophytes) | Valle San Lucano Flora, Agordo Fm. at Valle San Lucano, central Dolomites, Italy | [16] |
| Middle Triassic (Ladinian) | curvilinear leaf mine (DT222) | – | *Marattiopsis* sp. (marattialean pteridophyte) | Forcella da Cians/Ritberg Flora, Agordo Fm. near Wengen/La Valle, St. Veit-Seewald, within the Fernazza Fm., and St. Veit-Innerkohlbach, near Prags/Braies, within the Wengen/La Valle Fm., northern Dolomites, Italy | [16] |
| Late Triassic | linear mine | – | *Heidiphyllum elongatum* (Voltziales) | Cañadón Largo Fm., Argentina | [17] |
| Late Triassic (Carnian) | linear mine *Triassohypo-nomus dinmorenses* | Coleoptera | *Heidiphyllum elongatum* (Voltziales) | Blackstone Fm., Australia | [18,19] |
| **Stratigraphy** | **Ichno-species** | **Putative producer** | **Host plant** | **Localities** | **References** |
| Late Triassic (Carnian) | linear mine | Coleoptera | *Nilssonia sturri* (Cycadales) | Lunz Fm., Austria | [20] |
| Late Triassic (Middle Carnian) | mines parallel to the leaf margin | Coleoptera or Lepidoptera | *Cladophlebis nebbensis* (Filicales) | Momonoki Fm., Mine Group, Japan | [21] |
| Late Triassic (Carnian) | linear mines (DT41, DT71, and DT139) | Nematoceran fly (Diptera),  Symphyta (Hymenop-tera),  Polyphagan beetles (Coleoptera), or  basal Lepidoptera | *Heidiphyllum*  *elongatum* (Voltziales),  Ginkgoales, Cycadales, Umkomasia-les and Filicales | Molteno Fm., South  Africa | [22–25] |
| Late Triassic (Carnian) | *Cuniculonomus parallelus* (curvilinear mine) | polyphagan beetle (Coleoptera) or Lepidoptera | leaves of *Yabeiella wielandi* (Gnetales) | Potrerillos Fm. at the Quebrada del Durazno locality, Mendoza, Argentina | [26] |
| Late Triassic (Carnian) | *Foliomacula diffussus* (blotch mine) | Coleoptera, Xyelidae (Hymenop-tera) | *Heidiphyllum cacheutensis* (Voltziales) | Potrerillos Fm. at the Quebrada del Durazno locality, Mendoza, Argentina | [26] |
| Late Triassic (Ladinian–Carnian) | linear mines | – | undetermined plant host | Madygen Fm., Kyrgizstan | [27] |
| Late Triassic (Ladinian–Carnian) | linear mines | Coleoptera | *Heidiphyllum* sp. (Voltziales) | Santa María Fm., Brazil | [28] |

| **Stratigraphy** | **Ichno-species** | **Putative producer** | **Host plant** | **Localities** | **References** |
| --- | --- | --- | --- | --- | --- |
| early Permian (Kungurian) – Late Triassic (Anisian–Ladinian) | DT40, DT41, DT69, DT71, DT185, DT222 | – | *Scytophyllum bergeri*  (Pteridosper-males), ?*Pterophyllum* sp. (Cycadales), *Marattiopsis* sp.  (Marattiales) | Richthofen Fm., Agordo Fm., and Monte Agnello Flora, Italy | [29] |
| Late Triassic | linear mines | – | *Baiera multipartite* (Gingkophyta) | Upper Triassic Xujiahe Formation in Southwest China | [30] |

**Supplementary Table S2.** Studied material. Abbreviations: FG, collection TU Bergakademie Freiberg; Fm. Formation; MB, collection Museum of Natural History Berlin; NHMS, collection of Naturhistorisches Museum Schloss Bertholdsburg Schleusingen.

| **inventory number** | **type** | **host plant** | **locality** | **stratigraphy** |
| --- | --- | --- | --- | --- |
| MB.Pb.  1979/0004 | *A. maeandriformis* | *Autunia conferta* | Crock, Thuringia, Germany | Goldlauter Fm., early Permian, (Asselian) |
| MB.Pb.  1979/0012 | *A. maeandriformis* | *Autunia conferta* | Crock, Thuringia, Germany | Goldlauter Fm., early Permian, (Asselian) |
| MB.Pb.  1979/0013 | *A. maeandriformis* | *Autunia conferta* | Crock, Thuringia, Germany | Goldlauter Fm., early Permian, (Asselian) |
| MB.Pb.  1979/0014 | *A. maeandriformis* | *Autunia conferta* | Crock, Thuringia, Germany | Goldlauter Fm., early Permian, (Asselian) |
| MB.Pb.  1979/0015 | *A. maeandriformis* | *Autunia conferta* | Crock, Thuringia, Germany | Goldlauter Fm., early Permian, (Asselian) |
| MB.Pb.  1979/0020 | *A. maeandriformis* | *Autunia conferta* | Crock, Thuringia, Germany | Goldlauter Fm., early Permian, (Asselian) |
| MB.Pb.  1979/0044 | *A. maeandriformis* | *Autunia conferta* | Crock, Thuringia, Germany | Goldlauter Fm., early Permian, (Asselian) |
| MB.Pb.  1979/0069 | *A. maeandriformis* | *Autunia conferta* | Crock, Thuringia, Germany | Goldlauter Fm., early Permian, (Asselian) |
| MB.Pb.  1979/0181 | *A. maeandriformis* | *Autunia conferta* | Crock, Thuringia, Germany | Goldlauter Fm., early Permian, (Asselian) |
| MB.Pb.  1979/0182 | *A. maeandriformis* | *Autunia conferta* | Crock, Thuringia, Germany | Goldlauter Fm., early Permian, (Asselian) |
| MB.Pb.  1979/0184 | *A. maeandriformis* | *Autunia conferta* | Crock, Thuringia, Germany | Goldlauter Fm., early Permian, (Asselian) |
| MB.Pb.  1979/0349 | *A. maeandriformis* | *Autunia conferta* | Crock, Thuringia, Germany | Goldlauter Fm., early Permian, (Asselian) |
| MB.Pb.  1980/0517 | *A. maeandriformis* | *Autunia conferta* | Crock, Thuringia, Germany | Goldlauter Fm., early Permian, (Asselian) |
| MB.Pb.  2022/2423 | *A. maeandriformis* | *Autunia conferta* | Crock, Thuringia, Germany | Goldlauter Fm., early Permian, (Asselian) |
| 3500 | *A. maeandriformis* | *Autunia conferta* | Crock, Thuringia, Germany | Goldlauter Fm., early Permian, (Asselian) |

| **inventory number** | **type** | **host plant** | **locality** | **stratigraphy** |
| --- | --- | --- | --- | --- |
| MB.Pb.1979/0069 (thin section) | *A. maeandriformis* | *Autunia conferta* | Crock, Thuringia, Germany | Goldlauter Fm., early Permian, (Asselian) |
| MB.Pb.1979/0179 (thin section) | *A. maeandriformis* | *Autunia conferta* | Crock, Thuringia, Germany | Goldlauter Fm., early Permian, (Asselian) |
| MB.Pb.1979/0188 (thin section) | *A. maeandriformis* | *Autunia conferta* | Crock, Thuringia, Germany | Goldlauter Fm., early Permian, (Asselian) |
| FG 288/1 | *A. maeandriformis* | *Autunia conferta* | Crock, Thuringia, Germany | Goldlauter Fm., early Permian, (Asselian) |
| FG 288/4 | *A. maeandriformis* | *Autunia conferta* | Crock, Thuringia, Germany | Goldlauter Fm., early Permian, (Asselian) |
| FG 288/7 | holotype of *A. maeandriformis* Müller, 1982 | *Autunia conferta* | Crock, Thuringia, Germany | Goldlauter Fm., early Permian, (Asselian) |
| FG 288/19 | holotype of *A. divergens* Müller, 1982 | *Barthelop-teris germarii* | Homigtal near Breitenbach, Thuringia, Germany | Goldlauter Fm., early Permian, (Asselian) |
| NHMS Ap 37/5 | *A. maeandriformis* | *Autunia conferta* | Crock, Thuringia, Germany | Goldlauter Fm., early Permian, (Asselian) |

**Supplementary Table S3.** Abundance of leaf mining on *Autunia conferta* pinnae from several early Permian localities.

| **Locality** | **Total number of pinnules** | **Damaged pinnules** | **Undamaged pinnules** | **Uncertain damages** | **Content of damaged pinnules [%]** |
| --- | --- | --- | --- | --- | --- |
| Crock | 433 | 360 | 73 | 0 | 83.0 |
| Mittelberg | 29 | 0 | 29 | 1 | 0 |
| Oberhof | 96 | 10 | 87 | 1 | 11.5 |
| Sperbersbach, Schmücke | 18 | 0 | 18 | 2 | 0 |
| Gehlberg (Bahnhof) | 29 | 0 | 29 | 2 | 0 |
| Saar-Nahe Basin | 199 | 7 | 206 | 3 | 3.0 |
| Ottovice, Bohemia | 142 | 0 | 142 | 1 | 0 |

**Supplementary Note S1.** Micro-X-ray fluorescence (µXRF)

For investigations on the chemical composition of feeding traces and oviposition, we used an element distribution map of 1172 × 656 pixels, visualising the distribution of major and trace elements on the surface and immediate subsurface of a specimen from the Crock locality (MB.Pb.1979/0182). The image was processed by micro-X-ray fluorescence (µXRF) spectrometry using a Bruker M4 TORNADO PLUS µXRF spectrometer at MfN Berlin. Energy-dispersive Kα X-ray fluorescence maps were obtained from the sample at 2 mbar using a Rh X-ray tube focused by a polycapillary lens to a spot of ~20 µm on the sample surface. The X-ray tube was set to a maximum energy of 50 kV and 600 µA, a pixel resolution of 18 µm and dwell time per pixel of 50 ms was used to acquire the element map. The depth of focus of the X-ray optics was maximised by using the 1,000 µm aperture of the aperture management system incorporated into the Bruker Esprit operating system and the X-ray optics. Otherwise, no filters were used for data acquisition. Additional details of µXRF analysis of geological and palaeontological samples are given by [31–33].

Data visualisation was conducted using individual Kα X-ray fluorescence intensity maps. In addition, we extracted and quantified line profiles across two representative oviposition sites from the element distribution maps. Deconvolution of individual µXRF spectra in the line profiles and data quantification were carried out using a Fundamental Parameter approach [34,35] based on the Sherman equation [36] incorporated into the Bruker Esprit operating system. Elemental abundances were quantified as oxides and normalised to 100 wt%.

**Supplementary Note S2.** Results of micro-X-ray fluorescence spectrometry

Chemical element mapping by micro-X-ray fluorescence spectrometry (micro-XRF) of *Autunia conferta* leaves from the Crock locality revealed the chemical fingerprints of the sediment, the organically-preserved leaf compressions, and diagenetic overprint. Plant-insect interactions of *Asteronomus maeandriformis* leaf mines and oviposition are visualised by this method and can be clearly differentiated from the lamina by showing slightly different element distribution patterns. Most likely, these differences are related to the properties of their morphological relief on the lamina´s surface. It is worth noting that the chemical compositions are measured not only from the fossil's surface but also from the underlying sediment as the X-rays are infiltrating the specimen down to a few mm below the surface. Here, two specimens were chemically analysed (MB.Pb. 1979/0181, -/0182).

Chemical concentration maps of the following elements, measured as oxides, were analysed in detail: Al_2_O_3_, CaO, Cl, CoO, CuO, FeO, K_2_O, MgO, MnO, NO_2_, NiO, P_2_O_5_, SO_3_, SiO_2_, TiO_2_. The chemical differentiation between sedimentary matrix (clastic), organically-preserved leaf laminae, and post-depositional mineralisation is shown in the following.

Matrix:

**High**  Al_2_O_3_, K_2_O, MgO, NO_2_, CuO

**Moderate** CoO, MnO, NiO, P_2_O_5_, TiO_2_

**Low**  CaO, Cl, SO_3_, SiO_2_, FeO

The elemental composition of the matrix corresponds to the geochemistry of siliciclastic sediments (quartz, feldspars, mica, clay minerals). In addition, moderate or elevated concentrations of CoO, MnO, NiO, and P_2_O_5_ might reflect diagenetic overprint.

Leaf laminae:

**High** Cl, CoO (partly), CuO, FeO (partly), NiO, P_2_O_5_ (partly), SO_3_ (partly), TiO_2_

**Moderate** K_2_O, MnO, NO_2_,

**Low** Al_2_O_3_, CaO, MgO, SiO_2_,

The composition of the leaf laminae is referred to organic compounds as relicts of the original leaf biomass, on the one hand. Here, we see elevated concentrations of Cl and SO_3_ as organophilous agents. On the other hand, there is considerable diagenetic overprint manifested by increased concentrations of CoO, CuO, and FeO, and partly also SO_3_. These elevated element concentrations might be explained by post-sedimentary mineralisation processes during which sulfides were preferably precipitated in the acidic environment of the organic compounds.

A typical blocky pattern of the laminae is shown by the distribution of K_2_O, Cl, SO_3_, and SiO_2_, which reflects either the original structure of the mesophyll or the overall shrinkage of the organic matter. There is a considerable concentration of CoO, CuO, FeO, and SO_3_ in the leaf-central rachial region or midvein of the laminae where the organic matter is especially thick and the conducting tissue enabled hydrothermal fluid flow. The presence of sulfides in these specific regions is confirmed by thin sections. Even though having a similar chemical behaviour, NiO is not involved in these mineralisations but rather shows an affinity with the whole leaf-organic matter.

Diagenetic mineralisation:

**High** CaO, MnO, P_2_O_5_

**Moderate** -

**Low** Al_2_O_3_, Cl, CoO, CuO, FeO, K_2_O, MgO, NO_2_, NiO, SO_3_, SiO_2_, TiO_2_

The elemental composition most likely points to carbonate mineralisation during diagenesis. The absence of Mg in these areas is striking and points to aragonite or calcite, even though Mn and P are important agents, too, and might indicate the presence of mangano-calcite or accessory phosphates. Carbonate mineralisation occurred in tiny fissures of the fossil, but also as a coating of the cuticles, both adaxially and abaxially. The tiny cracks could have been formed by shrinkage of organic matter and later filled with carbonate.

**Element mapping of oviposition sites**

The chemical mapping revealed numerous oviposition sites of specimen MB.Pb.1979/0182 that are not all optically visible. They are characterised by a structural modification of the leaf surface at their margins (positive relief) visualised by higher concentrations of leaf compression-affiliated elements such as Cl and SO_3_, but also significantly increased concentrations of CoO and NiO (Fig. S2 e–g). These marginal structures represent the callus tissue around the insect eggs, which form a positive relief on the leaf surface and are constituted of organic compounds. Combined with a slightly increased SO_3_ concentration, the accumulation of these chalcophilous elements might be explained by a hydrothermal diagenetic event during which sulfide(?) minerals precipitated in the more acidic milieu of organic matter. In the center of the oviposition scars, increased concentrations of sediment-affiliated elements such as SiO_2_, MgO, and Al_2_O_3_ indicate a distinctly thinned lamina, suggesting that oviposition sites were causing a central depression in the lamina (Supplementary Fig. S3).

*A. maeandriformis* leaf mines of MB.Pb.1979/0182 show distinct structural modifications of the lamina with higher concentrations of Al_2_O_3_, SiO_2_, and MgO (Fig. S2 b–d), coinciding with lower concentrations of leaf-organic affiliated elements such as Cl and SO_3_. These element patterns clearly indicate a substantial loss of leaf-organic matter due to feeding activity. In contrast to oviposition scars, feeding traces do not show increased concentrations of CoO and NiO (Fig. S2 f–g). In addition, concentrations of NO_2_ seem to be slightly increased in the central region of the trace, which could be an indication of the hypothetical presence of digestive end products of the producers.

**Element mapping of leaf mines**

The scan provides insights into another leaf of *Autunia conferta* (MB.Pb.1979/0181), which shows several *Asteronomus maeandriformis* leaf mines and other interesting structures referred to plant-insect interactions.

The leaf mines are quite abundant and show a typical feeding pattern following the midvein of a pinnule and moving towards the main rachis of the pinna. The feeding traces are best visualised by elements of Cl, CuO, K_2_O, NiO, and SiO_2_. These element concentrations clearly show that the margins of the feeding traces exhibit abnormal thickening of the leaf-organic matter, most likely caused by reaction tissue formed during the lifetime of the plant. In contrast, the central furrow of the feeding traces shows a depletion of these elements, indicating partial loss of leaf-organic matter resulting from feeding. However, there is still organic matter present, suggesting that the external parts of the leaf (cuticle and epidermis) of the lamina have been unaffected by feeding. This is another indication pointing to the mining behaviour of the producers. In one feeding trace, a clear trend of increasing diameter from distal to basal lamina reflects the larval growth. At the end of the trace, the lamina clearly shows an oval hole that likely represents the escape position of the adult insect.

In addition, the pinnules show various spots where organophilous elements (e.g., Cl, SO_3_) are depleted and sediment-affiliated elements are enriched, pointing to a loss of leaf-organic matter. These spots represent round to slightly oval holes in the laminae, which are optically invisible. Similar structures have been recognised on other specimens of *A. conferta* and are interpreted to represent piercing and sucking structures produced by an unknown organism.

**Leaf mine with possible exit hole**

In the following, the focus is on the nearly complete leaf mine with a supposed escape structure. The chemical profile is shown in Suppl. Fig. S6. The following graphs show element distributions along the profile line. These graphs exhibit widely similar patterns to those in the graphs of profile line 1. In contrast to profile line 1, we see wider marginal areas of enriched organophilous elements and depleted sediment-affiliated elements. In the centre, there is a distinct peak of the sediment-affiliated elements and a depletion of the leaf-organic-affiliated. This distinct element-compositional trend reflects a nearly complete loss of leaf-organic matter in the elliptical central region of the feeding structure and is best explained to represent a hole in the lamina.

The same leaf mine is shown in in Suppl. Fig. S7. Most of the structures displayed by micro-XRF analysis were also detected with optical methods. The photograph in Suppl. Fig. S7a shows the terminal leaf mine with a widening extension that might be interpreted as a resting chamber of the larva while undergoing its final metamorphosis. The hole in the cuticle of the lamina could represent the escape structure after metamorphosis was finished.

Measurements on dimensions of the whole frass tunnel reveal insights to larval ontogenesis. The graph clearly shows a constant increase of mine width, thus reflecting constant growth of the larva (Fig. S7b). Interestingly, this trend is not disturbed by an interruption of the tunnel (see photograph), suggesting that this is not a true feeding interruption but only a change of the feeding direction in vertical orientation within the thickened lamina. The distinctly increased diameter of the terminal tunnel reflects the dimensions of the widened resting chamber.

**Concluding remarks**

In conclusion, chemical element maps provide important insights to the fossil nature and taphonomic pathways of leaf compressions (*Autunia conferta*) from the early Permian Crock locality. The results show a clear distinction of the fossil organic matter from the embedding sedimentary rocks. In addition, the methodology reflects the morphological properties of arthropod feeding traces (*Asteronomus maeandriformis*) and associated oviposition sites. The main result is the proof of these structures as being of fossil origin but also providing information on their morphological relief and accompanied modification of the original leaf laminae, which are optically poorly or nearly invisible.

**Supplementary Note S3.** Statistical analysis

In total, we measured lengths and widths of 320 oviposition scars from 17 specimens. Additionally, we determined dimensions of five insect egg remains (Fig. S8).

The frequency distribution functions in Supplementary Figs. S8 b, c revealed normally distributed scar dimensions, but some scars are outliers and likely belong to other morphotypes. Therefore, we applied the 1.5 interquartile range (IQR) rule to exclude outliers from the dataset [37] and determined lower and upper quartiles (Q1, Q3) of the data. According to the IQR rule outliers of a dataset either fall below Q1-1.5 IQR or above Q3+1.5 IQR (Suppl. Fig. S8c).

In total, we found 288 scars within the interquartile range of lengths between 0.53 mm and 1.24 mm and within the interquartile range of widths between 0.25 mm and 0.80 mm. Consequently, these scars most likely belong to the same morphotype. Furthermore, 38 oviposition scars we identified as outliers and excluded them from the dataset.

Statistical analysis of the dataset cleaned of outliers (n=288) revealed the following results:

|  | lower limit (mm) | upper limit (mm) | mean value  (mm) | standard deviation σ |
| --- | --- | --- | --- | --- |
| scar lengths | 0.59 | 1.21 | 0.87 | 0.12 |
| scar widths | 0.34 | 0.78 | 0.52 | 0.08 |

Additionally, we measured the widths of 33 leaf mines at different positions (Figure S9).


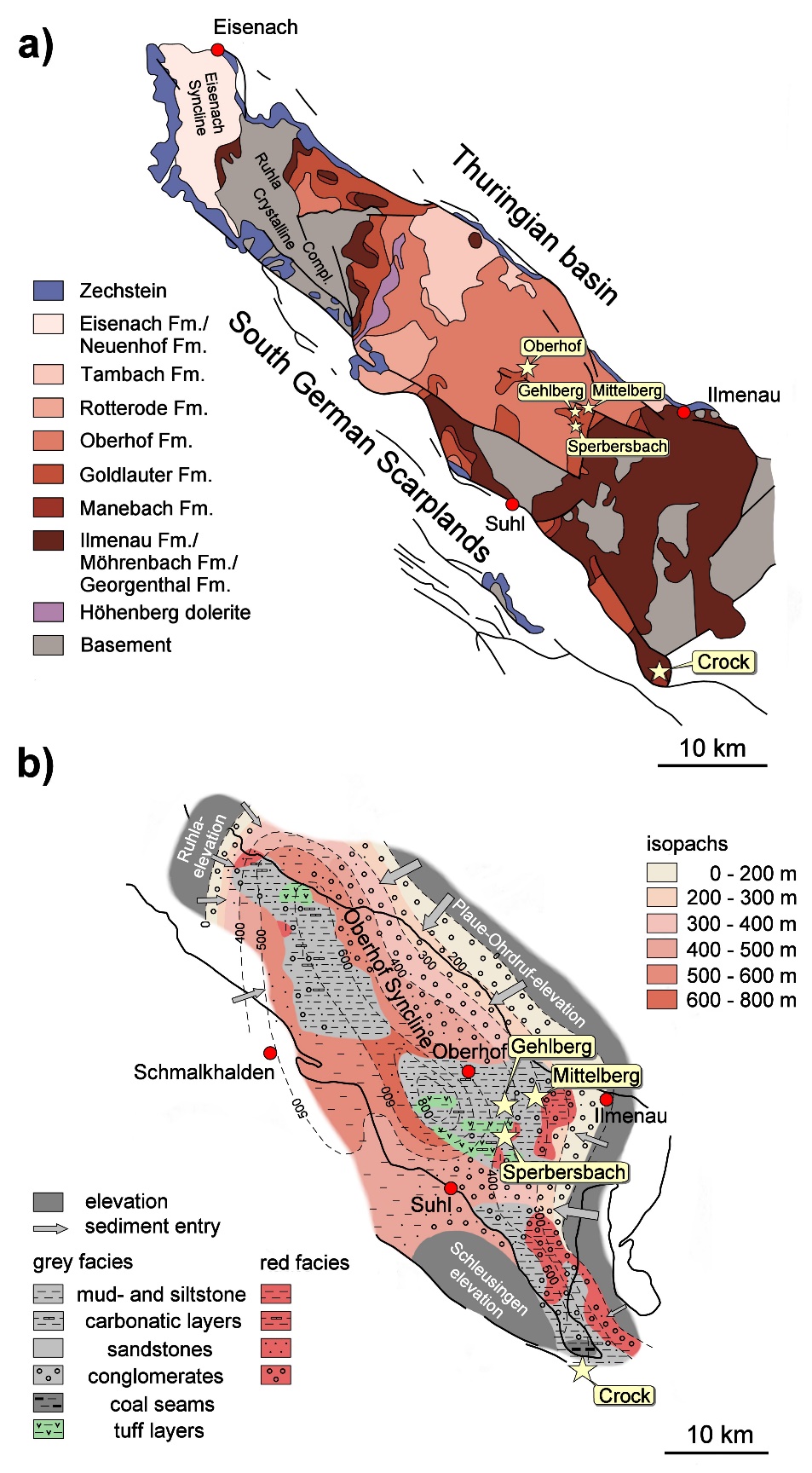


**Supplementary Figure S1.** Geological map of the Thuringian Forest Basin in central Germany. (a) Simplified geological map of the Thuringian Forest Basin and location of the locality Crock. Adapted from [38,39]. (b) Palaeogeographic and facies map of the Thuringian Forest Basin at the age of the lower Goldlauter Formation. Adapted from [38,40].


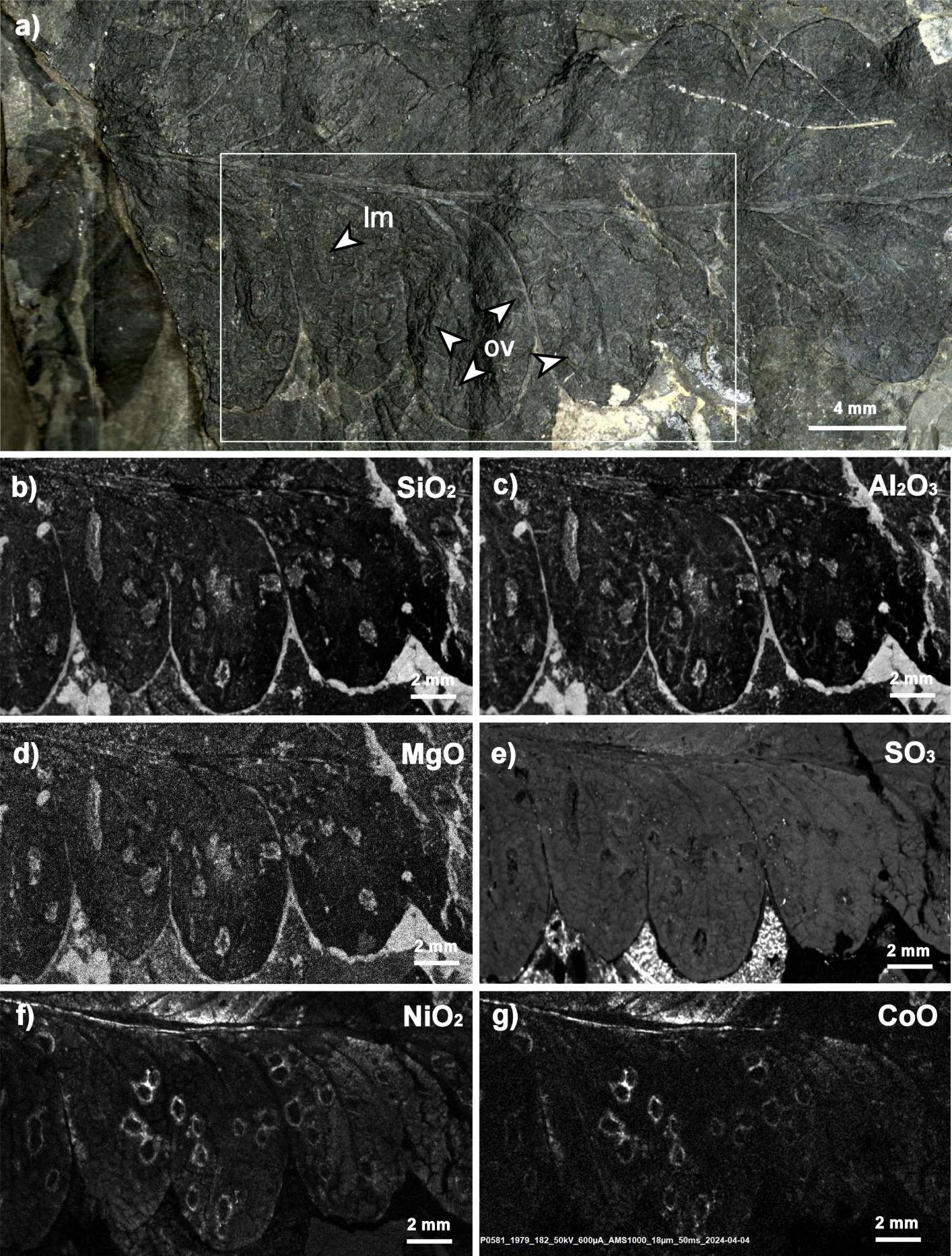


**Supplementary Figure S2.** Element mapping of *Autunia conferta* with oviposition sites by micro-X-ray fluorescence spectrometry (micro-XRF). (a) A stitched panorama photograph of the specimen shows the section where elements were mapped. Abbreviations: lm – *A. maeandriformis* leaf mine; ov – oviposition sites. (b–g) Chemical maps of corresponding elements in grey scales: Bright colours indicate high concentrations, whereas dark colours indicate low concentrations.


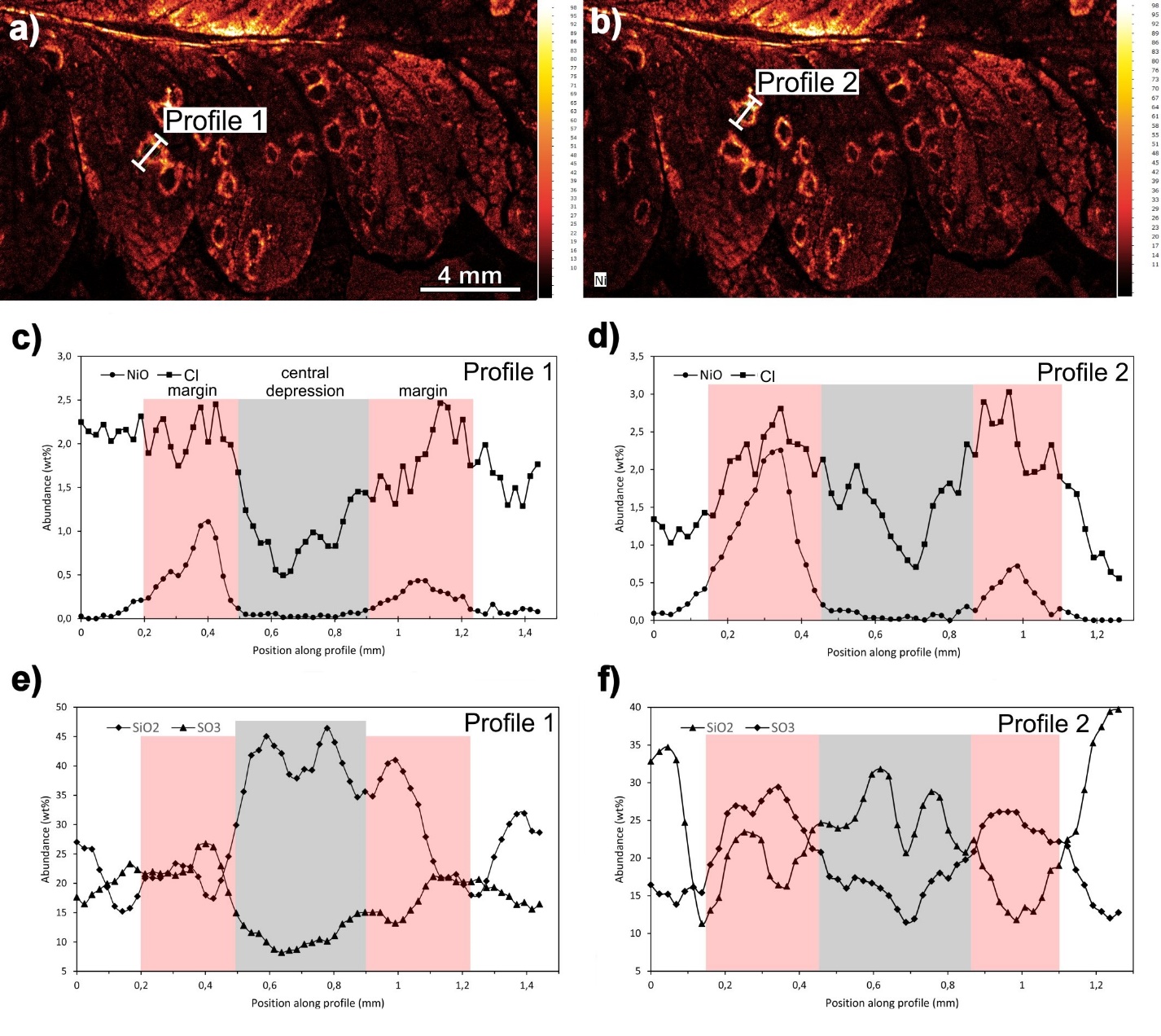


**Supplementary Figure S3.** Element distribution in selected oviposition scars in *Autunia conferta*. (a, b) Positions of profiles 1 and 2. (c, e) Graphical presentation of selected elements along the transects of profile 1. The red and grey regions show the assumed position of the callus tissue (red) with increased concentrations of NiO, Cl, and SO_3_ and of the central depression (grey) with elevated concentrations of SiO_2_ but reduced concentrations of SO_3_. (d, f) The same information was provided for Profile 2.

**
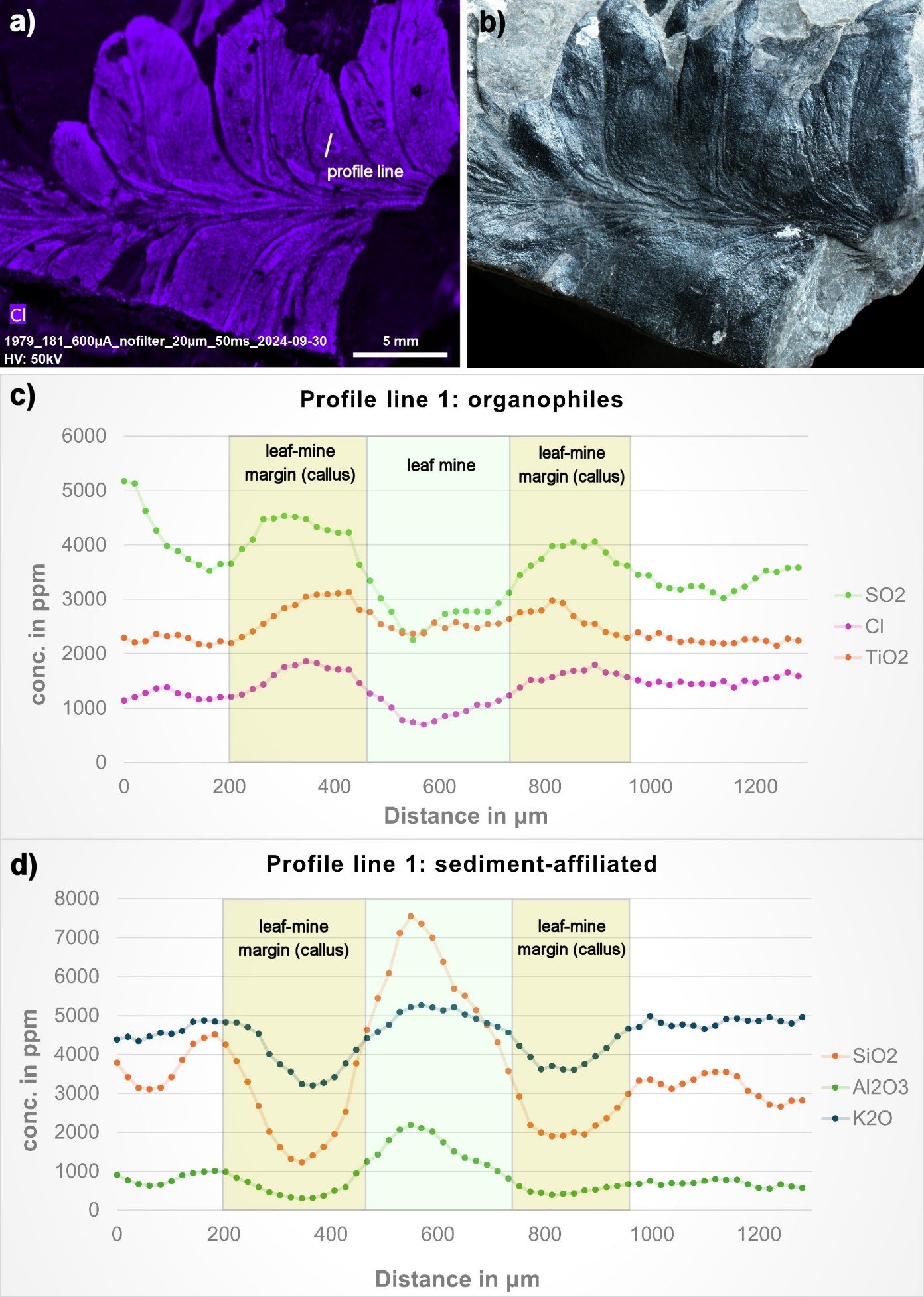
**

**Supplementary Figure S4.** Element distribution in different regions of *Asteronomus maeandriformis*. (a) XRF image (chlorine) of specimen MB.Pb.1979/0181 (b) Photograph of the specimen figured in (a). (c) Distribution of organophiles along the profile line figured in (a). (d) Distribution of sediment-affiliated elements along the profile line figured in (a).


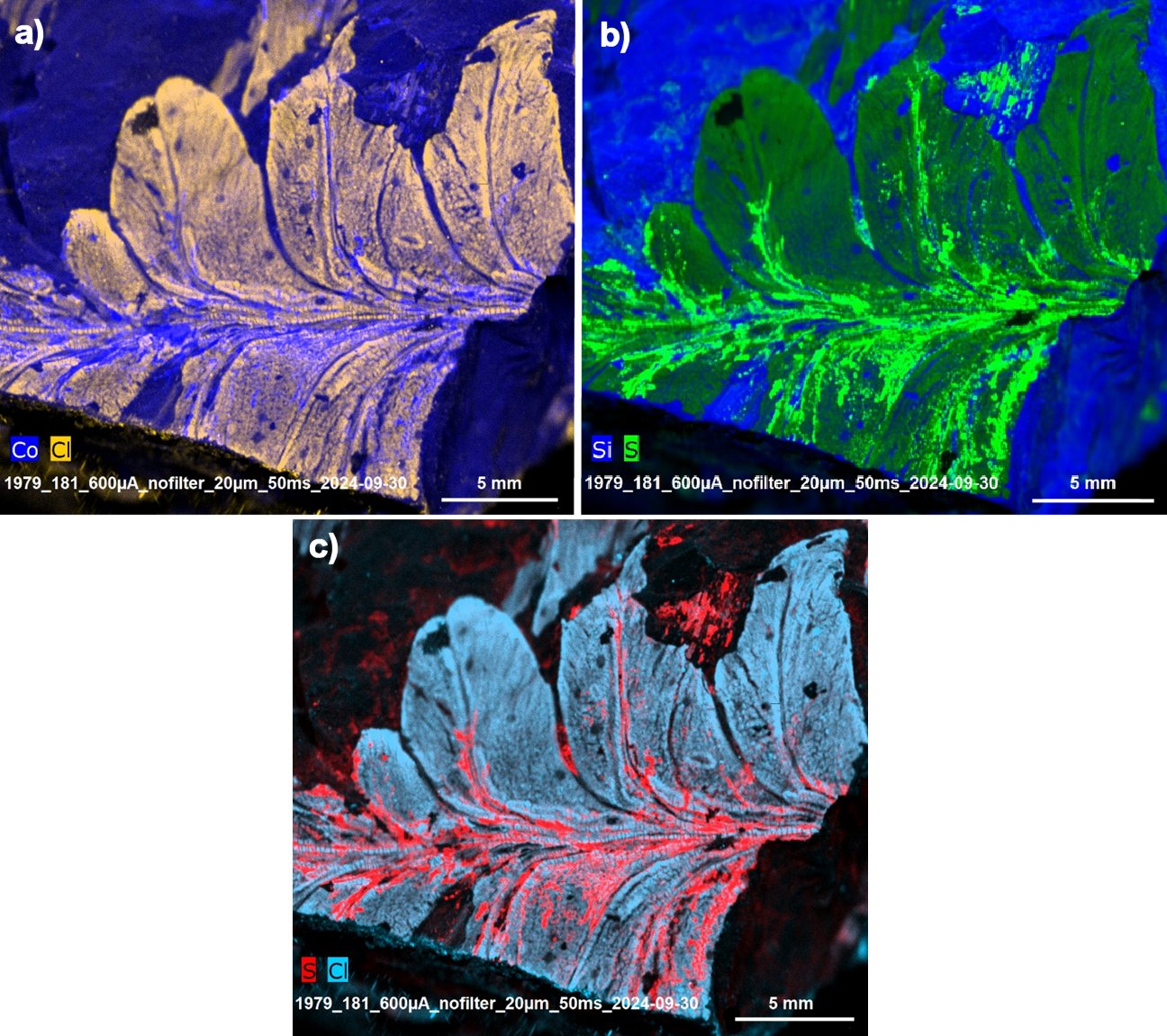


**Supplementary Figure S5.** Combined chemical element maps of specimen MB.Pb.1979/181. Note that there is no correlation between sulfidic elements (CoO, SO_3_) and element tracers of leaf organic matter (Cl), and not with the sediment (SiO2). CoO and SO_3_ do not show any structures within the mines pointing to the presence of coprolites.

**
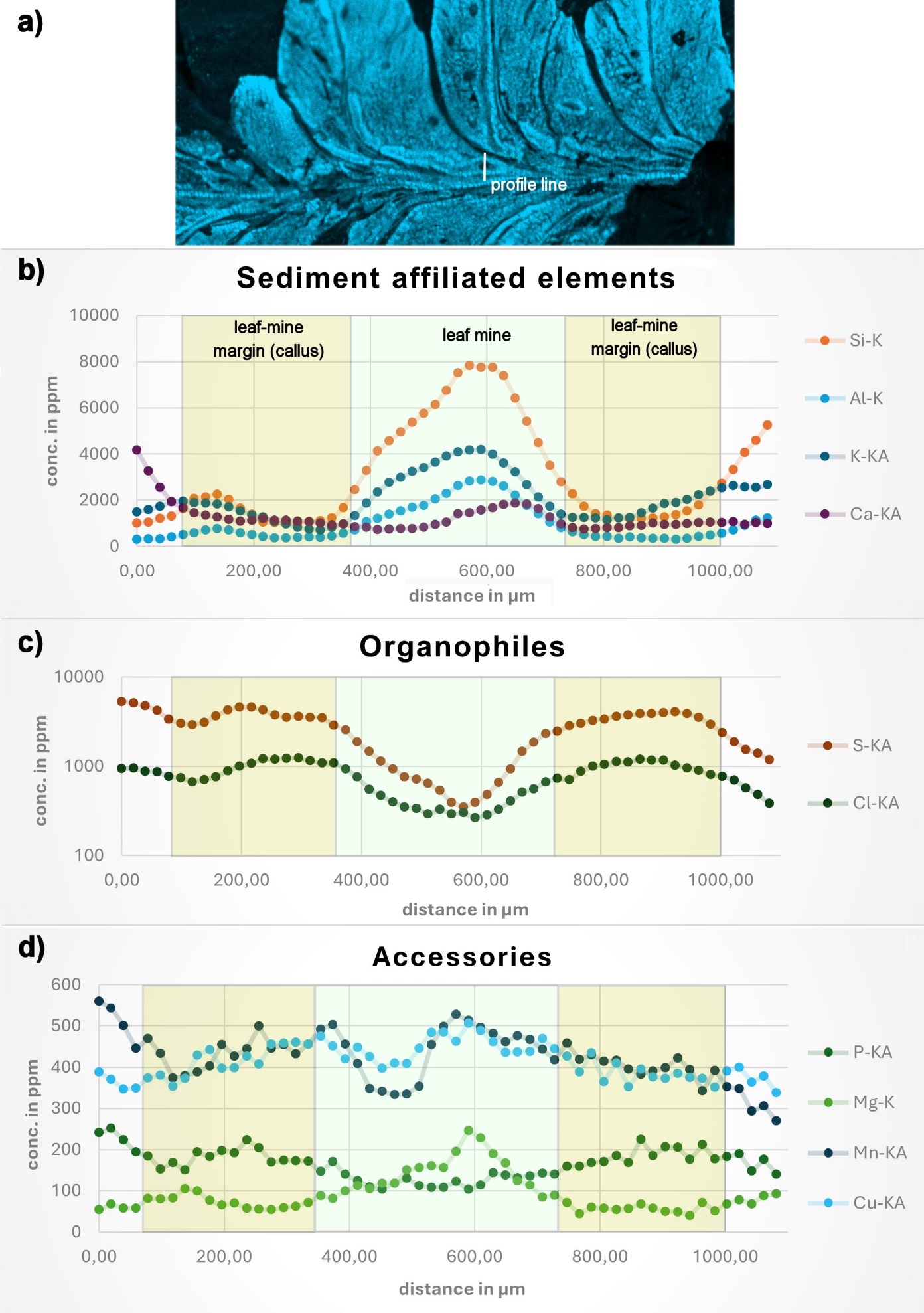
**

**Supplementary Figure S6.** Element distribution in selected areas of *Asteronomus* leaf mines. (a) micro-XRF image of specimen MB.Pb.1979/0181 showing the profile line of the following graphs. (b) Distribution of elements in the sediment along the profile line in (A). (c) Distribution of organophiles along the profile line in (A). (d) Content of accessories along the profile line in (a).

**(a)**


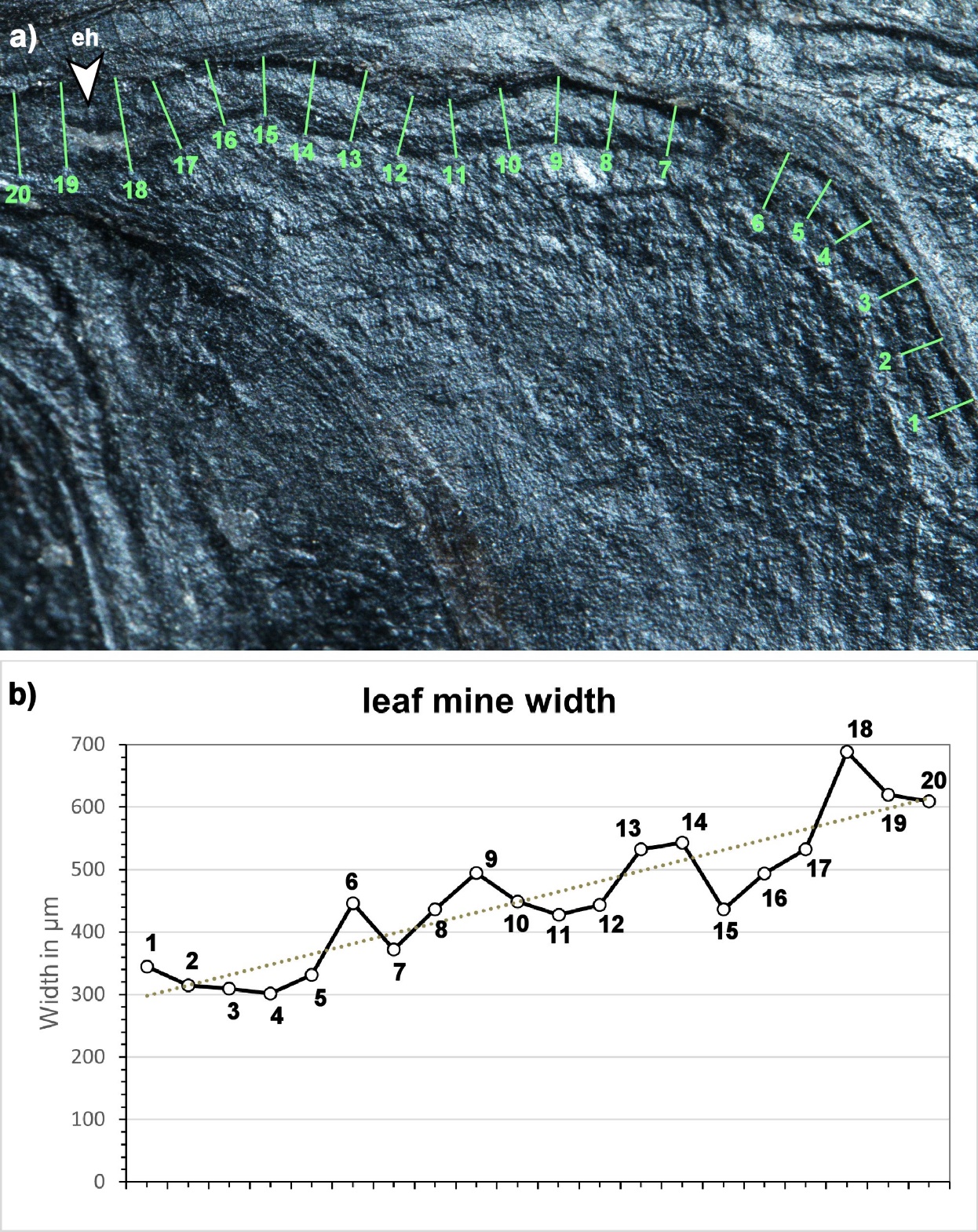


**Supplementary Figure S7.** Measurements of tunnel widths of an *A. maeandriformis* leaf mine with putative exit hole. (a) Photograph of specimen MB.Pb.1979/0181 showing the positions (green) where mine widths were measured. eh – putative exit hole. (b) Leaf mine widths measured from proximal to distal near the midvein.


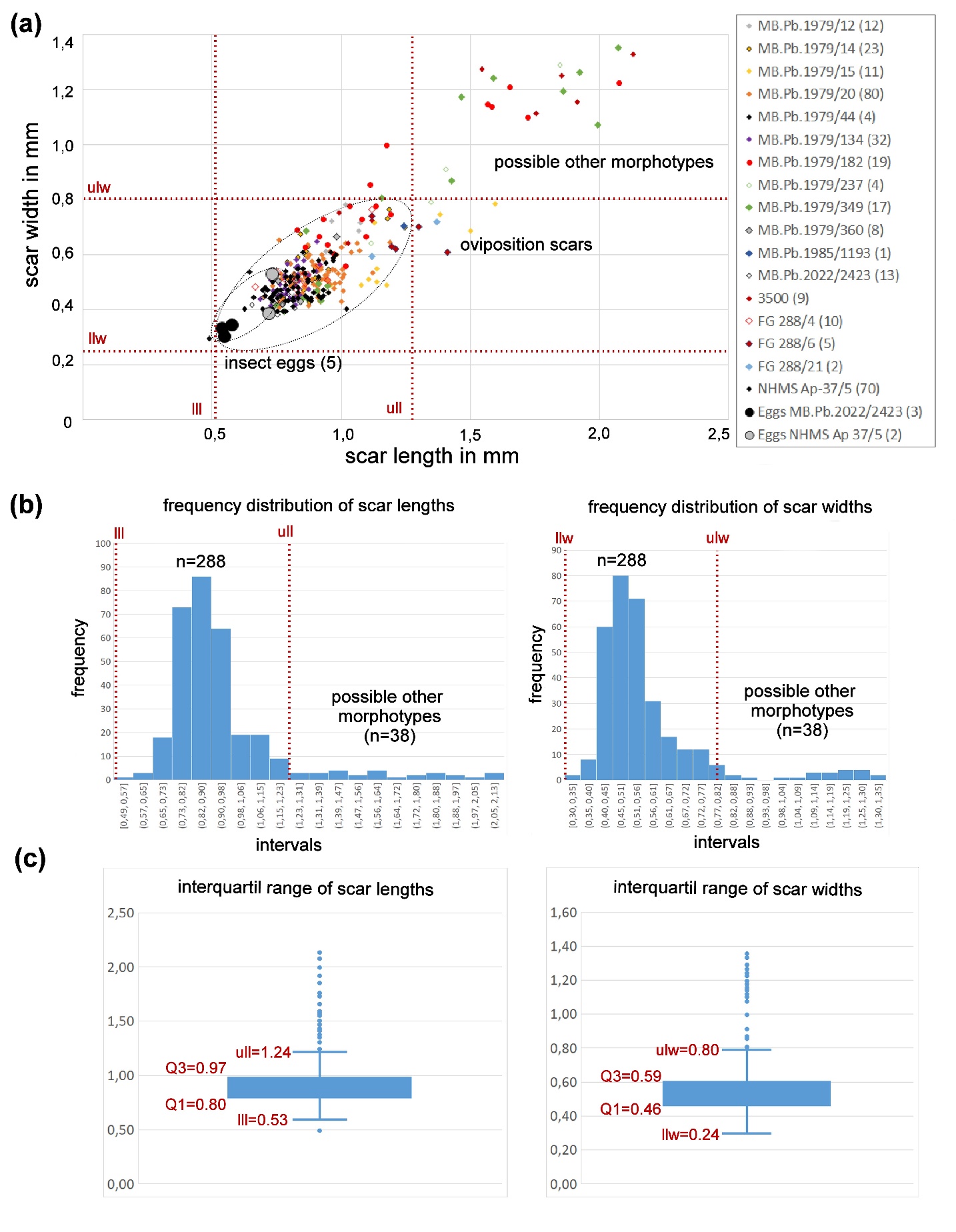


**Supplementary Figure S8.** Size variation of oviposition scars and insect eggs of *Asteronomus maeandriformis*. (a) Measurements of oviposition scars and insect egg impressions on *Autunia conferta*. (b) Frequency distribution of oviposition lengths and widths. (c) Results of the 1.5 interquartile range (IQR) rule. Abbreviations: Q1, lower quartile; Q3, upper quartile; n, number of scars; lll, lower limit of scar lengths; ull, upper limit of scar lengths; llw, lower limit of scar widths; ulw, upper limit of scar widths.


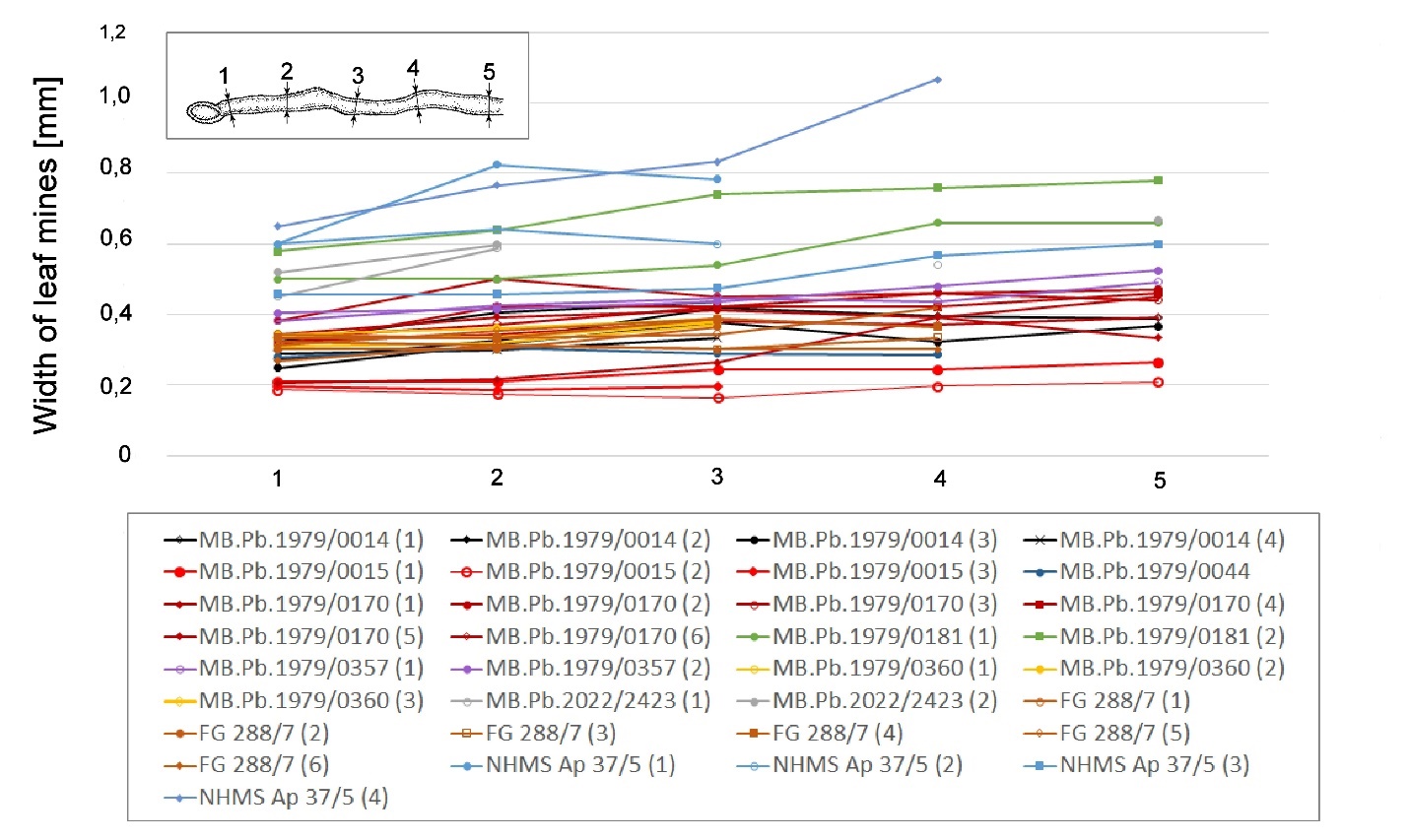


**Supplementary Figure S9.** Leaf mine widths along the frass trails. Note that most leaf mines became wider towards the midrib. Measurements were taken from scaled figures.


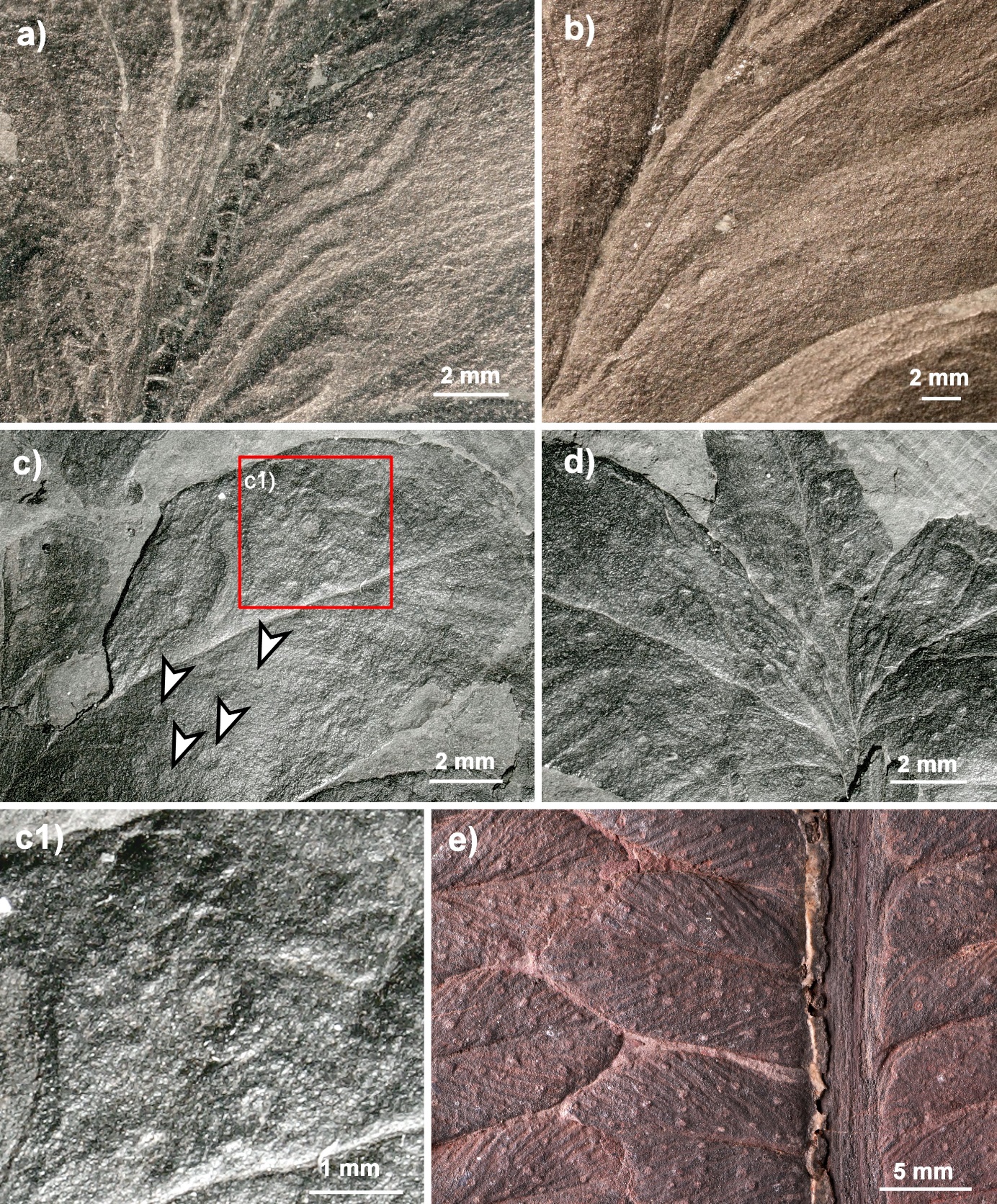


**Supplementary Figure S10.** Co-occurrence of piercing and sucking and *Asteronomus* on *Autunia conferta*. (a) *A. maeandriformis* (MB.Pb.1979/0004). (b) Piercing and sucking and oviposition on the same specimen depicted in (a). (c, d) Another example for the co-occurrence of piercing and sucking (c1 and arrows) and *A. maeandriformis* (MB.Pb.1979/0184). Note the reaction tissue bordering the punctures. (e) Putative secretory cavities on *Autunia conferta* from the Saar-Nahe Basin (MB.Pb. xxx).

**Supplementary** **References**

1. Knecht, R. J. et al. Endophytic ancestors of modern leaf miners may have evolved in the Late Carboniferous. New Phytol. **240,** 2050–2057 (2023).

2. Müller, A. H. Über Hyponyme fossiler und rezenter Insekten, erster Beitrag. Freiberg. Forsch. H. C **366,** 7–27 (1982).

3. Müller, A. H. Allgemeine Grundlagen. 5th ed. (Verlag nicht ermittelbar, 1992).

4. Castro, M. P. Huellas de actividad biológica sobre plantas del Estefaniense superior de La Magdalena (León, España). Revista Española de Paleontología **12,** 52–66 (1997).

5. Laaß, M. & Hauschke, N. First evidence of borings in calamitean stems and other plant-arthropod interactions from the late Pennsylvanian of the Saale Basin; 10.25673/90941 (2019).

6. Laaß, M. & Rößler, R. in Die Rotliegend-Fauna des Thüringer Waldes, edited by R. Werneburg & J. W. Schneider (NaturHistorisches Museum Schloss Bertoldsburg, 2024), pp. 115–125.

7. Potonié, H. Die Flora des Rothliegenden von Thüringen. Abh. Königl. Preuss. Geol. Landesanstalt n. F. **9** (1893).

8. Potonié, R. Mitteilungen über mazerierte kohlige Pflanzenfossilien. Z. Bot. **13,** 79–88 (1921).

9. Labandeira, C. C. & Beall, B. S. in Arthropod paleobiology, edited by D. G. Mikulic (1990), pp. 214–256.

10. Adami-Rodrigues, K., Iannuzzi, R. & Pinto, I. D. in Trace fossils in evolutionary palaeoecology, edited by B. D. Webby, M. G. Mangano & L. A. Buatois (Taylor & Francis, 2004), pp. 106–125.

11. Souza Pinheiro, E. R. de, Iannuzzi, R. & Tybusch, G. P. Specificity of leaf damage in the Permian “Glossopteris Flora”: A quantitative approach. Review of Palaeobotany and Palynology **174,** 113–121 (2012).

12. Cariglino, B. Patterns of insect-mediated damage in a Permian Glossopteris flora from Patagonia (Argentina). Palaeogeogr., Palaeoclimatol., Palaeoecol. **507,** 39–51 (2018).

13. Zhou, W.-M., Wan, M.-L., Pšenička, J. & WANG, J. U. Discovery of coprolites in an Early Permian fern mesophyll. PE **5** (2022).

14. Krassilov, V. & Karasev, E. First evidence of plant – arthropod interaction at the Permian – Triassic boundary in the Volga Basin, European Russia. Alavesia **2,** 247–252 (2008).

15. Adami-Rodrigues, K., Barboni, R., Dutra, T. L. & Wilberger, T. in Congresso Brasileiro de Paleontologia (2011), pp. 348–354.

16. Labandeira, C. C., Kustatscher, E. & Wappler, T. Floral Assemblages and Patterns of Insect Herbivory during the Permian to Triassic of Northeastern Italy. PloS one **11,** e0165205 (2016).

17. Adami-Rodrigues, K., Gnaedinger, S. & Gallego, O. in XII Simpósio de Paleobotânicos e Palinólogos, Resumo (2008), p. 1.

18. Rozefelds, A. C. & Sobbe, I. Problematic insect leaf mines from the Upper Triassic Ipswich Coal Measures of southeastern Queensland, Australia. Alcheringa: An Australasian Journal of Palaeontology **11,** 51–57 (1987).

19. Tillyard, R. J. Mesozoic Insects of Queensland. No. 9. Orthoptera, and additions to the Prolorthoptera, Odonata, Hemiptera and Planipennia. Proceedings of the Linnean Society of New South Wales **47,** 447–470 (1922).

20. Meller, B., Ponomarenko, A. G., Vasilenko, D. V., Fischer, T. C. & Aschauer, B. First beetle elytra, abdomen (Coleoptera) and a mine trace from Lunz (Carnian, Late Triassic, Lunz‐am‐See, Austria) and their taphonomical and evolutionary aspects. Palaeontology **54,** 97–110 (2011).

21. Imada, Y., Oyama, N., Shinoda, K., Takahashi, H. & Yukawa, H. Oldest leaf mine trace fossil from East Asia provides insight into ancient nutritional flow in a plant-herbivore interaction. Sci. Rep. **12,** 5254 (2022).

22. Scott, A. C., Anderson, J. M. & Anderson, H. M. Evidence of plant–insect interactions in the Upper Triassic Molteno Formation of South Africa. JGS **161,** 401–410 (2004).

23. Labandeira, C. C. in Insect Outbreaks Revisited, edited by P. Barbosa, D. K. Letourneau & A. A. Agrawal (Wiley, 2012), pp. 267–290.

24. Labandeira, C. C., Anderson, J. M. & Anderson, H. M. in The Late Triassic World, edited by L. H. Tanner (Springer International Publishing, 2018), pp. 623–719.

25. Labandeira, C. C. & Anderson, J. M. in Geological Society of America Abstracts with Programs (2005), p. 15.

26. Cariglino, B., Prevec, R., Lara, M. B. & Zavattieri, A. M. Evidence of insect leaf mining on Late Triassic (Carnian) gymnosperms from Argentina. Rev. Palaeobot. Palynol. **305,** 104749 (2022).

27. Vasilenko, D. V. in Modern Paleontology: Classical and New Methods (2009), pp. 9–15.

28. Cenci, R., Adami-Rodrigues, K. & Urban, C. in XXIII Congreso Brasileiro de Paleontologia, Resumo (2013), p. 302.

29. Wappler, T., Kustatscher, E. & Dellantonio, E. Plant-insect interactions from Middle Triassic (late Ladinian) of Monte Agnello (Dolomites, N-Italy)-initial pattern and response to abiotic environmental perturbations. PeerJ **3,** e921 (2015).

30. hao, T., Wan, S., Li, S. & Feng, Z. Leaf mining induced chemical defense of a Late Triassic ginkgophyte plant. The New phytologist; 10.1111/nph.20154 (2024).

31. Vansteenberge, S. et al. Benchtop μXRF as a tool for speleothem trace elemental analysis: Validation, limitations and application on an Eemian to early Weichselian (125–97 ka) stalagmite from Belgium. Palaeogeography, Palaeoclimatology, Palaeoecology **538,** 109460 (2020).

32. Kaskes, P., Déhais, T., Graaff, S. J. de, Goderis, S. & Claeys, P. in Large Meteorite Impacts and Planetary Evolution VI, edited by W. U. Reimold & C. Koeberl (Geological Society of America, 2021), pp. 171–206.

33. Winter, N. J. de & Claeys, P. Micro X‐ray fluorescence (μ XRF ) line scanning on Cretaceous rudist bivalves: A new method for reproducible trace element profiles in bivalve calcite. Sedimentology **64,** 231–251 (2017).

34. Rousseau, R. M. Fundamental algorithm between concentration and intensity in XRF analysis 2—practical application. X-Ray Spectrometry **13,** 121–125 (1984).

35. Rousseau, R. M. Fundamental algorithm between concentration and intensity in XRF analysis 1—theory. X-Ray Spectrometry **13,** 115–120 (1984).

36. Sherman, J. The theoretical derivation of fluorescent X-ray intensities from mixtures. Spectrochimica Acta **7,** 283–306 (1955).

37. Dekking, F. M., Kraaikamp, C., Lopuhaä, H. P. & Meester, L. E. A Modern Introduction to Probability and Statistics (Springer London, 2005).

38. Lützner, H., Andreas, D., Schneider, J. W., Voigt, S. & Werneburg, W. in Stratigraphie von Deutschland X. Rotliegend. Teil I: Innervariscische Becken, edited by H. Lützner & G. Kowalczyk (2012), pp. 418–487.

39. Schneider, J. W. et al. in Die Rotliegend-Fauna des Thüringer Waldes, edited by R. Werneburg & J. W. Schneider (Semana-Sonderveröffentlichung, 2024), pp. 199–256.

40. Haubold, H. Stratigraphische Grundlagen des Stefan C und Rotliegenden im Thüringer Wald. Schriftenr. Geol. Wiss. Berlin **23,** 1–110 (1985).
